# Age-associated remodeling of NK cell subsets, B cell subsets, and T cell surface markers in peripheral blood of healthy Chinese adults: a prospective multicolor flow cytometry study

**DOI:** 10.3389/fragi.2026.1847033

**Published:** 2026-10-09

**Authors:** Liyuan Zheng, Wei Cao, Yixuan Wang, Tengda Xu, Fada Wang, Leidan Zhang, Xinchao Liu, Taisheng Li

**Affiliations:** 1 Department of Infectious Diseases, Peking Union Medical College Hospital, Chinese Academy of Medical Sciences and Peking Union Medical College, Beijing, China; 2 Health Management Center, Peking Union Medical College Hospital, Chinese Academy of Medical Sciences and Peking Union Medical College, Beijing, China; 3 State Key Laboratory for Complex, Severe, and Rare Diseases, Peking Union Medical College Hospital, Chinese Academy of Medical Sciences and Peking Union Medical College, Beijing, China; 4 Center for AIDS Research, Chinese Academy of Medical Sciences, Beijing, China

**Keywords:** B cell subsets, Chinese population, healthy adults, immunosenescence, multicolor flow cytometry, NK cell subsets, TEMRA

## Abstract

**Background:**

Immunosenescence refers to the structural and functional remodeling of the immune system that accompanies aging, and is closely linked to increased susceptibility to infection, diminished vaccine responses, and a higher burden of chronic disease. While flow cytometric profiling of peripheral blood lymphocyte subsets has become a key tool for characterizing immune aging, systematic age-related data on NK cell subsets (CD56hi/CD56dim/CD56neg three-way classification) and B cell subsets (IgD/CD27 four-way classification) in healthy Chinese adults remain scarce. Quantitative age trends for CD57 and TEMRA as markers of terminal T cell differentiation have also been incompletely described in this population.

**Methods:**

We prospectively enrolled 146 healthy adults attending the annual health examination center of Peking Union Medical College Hospital (Panel 1: n = 103, age 20–82 years; Panel 2: n = 43, age 25–92 years, enriched for older participants). Panel 1 was a 12-color single-tube assay covering T cells (including CD57), NK cell trisubsets, and IgD/CD27-based B cell subsets. Panel 2 was a 12-color dual-tube assay focused on detailed T cell differentiation, incorporating CD45RA/CD62L, CD28, CD31, CD38, and HLA-DR. Spearman rank correlation, Kruskal–Wallis, and Mann-Whitney U tests were used for statistical analysis.

**Results:**

In Panel 1 (n = 103), ten parameters showed significant age correlations. CD4^+^CD57^+^ (*r* = +0.421, p < 0.001) and CD8^+^CD57^+^ (*r* = +0.338, p < 0.001) rose most strongly with age. Total NK frequency increased (*r* = +0.259, p = 0.008) while CD56hiCD16^−^ regulatory NK cells declined (*r* = −0.263, p = 0.007) and NK PD-1 expression rose (*r* = +0.256, p = 0.009). IgD^+^CD27^+^ marginal zone-like B cells declined significantly in Panel 1 (*r* = −0.257, p = 0.009). Panel 2 (n = 43) revealed the strongest signals in CD8^+^ T cells: TEMRA accumulation (*r* = +0.532), CD28 loss (*r* = −0.552), and naive depletion (*r* = −0.534) all reached p < 0.001. CD4^+^ naive cells also declined significantly (*r* = −0.303, p = 0.048). Multivariate analysis confirmed that most associations were independent of sex.

**Conclusion:**

This study provides a comprehensive characterization of age-related immunophenotypic changes across NK cell subsets, IgD/CD27-defined B cell subsets, and multidimensional T cell markers in healthy Chinese adults, offering reference data for clinical immune assessment and vaccine response prediction.

## 1 Introduction

Aging is one of the most consistent drivers of immune dysfunction across species. In humans, the progressive remodeling of both innate and adaptive immunity that accompanies aging—collectively termed immunosenescence—underlies much of the increased susceptibility to infection, poor vaccine immunogenicity, and impaired tumor surveillance seen in older individuals (Montecino-Rodriguez et al., 2013; Nikolich-Žugich, 2018). These changes are not merely quantitative; they reflect deep shifts in the composition and functional capacity of circulating lymphocyte populations, and are now recognized as a contributing factor in a range of age-related diseases and geriatric syndromes (Franceschi et al., 2018; GBD 2017 Mortality Collaborators, 2018).

Multicolor flow cytometry has made it possible to capture these shifts in considerable detail. Rather than simply counting T, B, and NK cells, it is now routine to interrogate differentiation state, activation status, and functional marker expression all at once within the same sample (Maecker et al., 2012). From this work, a fairly consistent picture has emerged for T cells: the loss of CD28 co-stimulation, the accumulation of CD57-expressing terminally differentiated cells, and the build-up of TEMRA (terminally differentiated effector memory cells re-expressing CD45RA) are now considered hallmarks of T cell aging (Fülöp et al., 2013; Weng, 2006). CD31 (PECAM-1) deserves particular mention as a marker of recent thymic emigrants (RTEs) — its decline with age is a direct readout of thymic involution and reduced naive T cell output (Ferrando-Martínez et al., 2011). For NK cells, the picture is one of functional rebalancing: the immunoregulatory CD56^hi^CD16^−^ subset contracts with age, while the cytotoxic CD56^dim^CD16^+^ fraction becomes proportionally dominant (Solana et al., 2012; Hazeldine and Lord, 2013). The atypical CD56^−^CD16^+^ subset has attracted attention in the context of chronic infection and may also expand during normal aging (Solana et al., 2012). In the B cell compartment, the IgD/CD27 four-subset framework has been useful for capturing age-related contraction of switched memory B cells and shifts in the double-negative (IgD^−^CD27^−^) population, sometimes referred to as age-associated B cells (ABCs) (Frasca and Blomberg, 2014; Rubtsov et al., 2011).

Despite the richness of this literature, systematic data from healthy Chinese adults are still relatively sparse (Qin et al., 2016; Chang et al., 2024), and an integrated analysis covering NK cell trisubsets, the IgD/CD27 B cell framework, and terminal T cell differentiation markers (CD57 and TEMRA) in this population has not previously been reported. We therefore designed two complementary multicolor flow cytometry panels and applied them to 146 healthy adults spanning 20–92 years of age, with the goal of building an age-stratified reference dataset for peripheral blood immunophenotyping in the Chinese population.

## 2 Materials and methods

### 2.1 Participants

Participants were recruited prospectively between 2023 and 2024 from the annual health examination center of Peking Union Medical College Hospital (PUMCH). Eligibility required age ≥18 years, no clinically significant abnormalities on routine examination, and written informed consent. We excluded individuals with known autoimmune disease, chronic infections (including HIV, HBV, or HCV), hematological disorders, recent use of immunosuppressive agents, glucocorticoids, or biologics (within 3 months), acute infection in the preceding 4 weeks, pregnancy or lactation, or evidence of malignancy or severe organ dysfunction. The study was approved by the PUMCH Institutional Review Board (ethics number: I-24PJ0474).

A total of 146 participants were enrolled across two non-overlapping cohorts. Panel 1 (n = 103) comprised 86 men and 17 women, age range 20–82 years (mean ± SD: 45.3 ± 14.0 years). Panel 2 (n = 43) comprised 29 men and 14 women, age range 25–92 years (mean ± SD: 63.6 ± 19.8 years); this cohort was intentionally enriched for older participants to improve statistical power for detecting age effects at the upper end of the age spectrum. Demographic characteristics are summarized in Table 1.

**TABLE 1:** Participant demographics.

Characteristic	Panel 1 (n = 103)	Panel 2 (n = 43)
Age, mean ± SD (years)	45.3 ± 14.0	63.6 ± 19.8
Age range (years)	20–82	25–92
Median age (years)	44	70
Male, n (%)	86 (83.5%)	29 (67.4%)
Female, n (%)	17 (16.5%)	14 (32.6%)
Young group (20–44 years), n (%)	52 (50.5%)	11 (25.6%)
Middle-aged group (45–64 years), n (%)	39 (37.9%)	5 (11.6%)
Older group (≥65 years), n (%)	12 (11.7%)	27 (62.8%)

SD, standard deviation.

### 2.2 Sample processing

Fasting peripheral venous blood (5 mL) was collected into EDTA-anticoagulated tubes in the morning and processed within 2 h. For surface staining, 200 μL of whole blood was incubated with fluorochrome-conjugated antibodies at room temperature in the dark for 20 min, followed by red blood cell lysis with BD FACS Lysing Solution (1:10 dilution, 10 min at room temperature), centrifugation, and two PBS washes. Intracellular Ki67 staining was performed using the BD Cytofix/Cytoperm kit per manufacturer’s instructions, with anti-Ki-67 (BV421) added after fixation and permeabilization (30 min incubation), followed by washing and immediate acquisition.

### 2.3 Flow cytometry panels

All samples were acquired on a BD LSRFortessa, with data collection *via* BD FACSDiva software and analysis in FlowJo v10 (BD Biosciences).

Panel 1 was a 12-color single-tube assay (n = 103) designed to cover T, NK, and B cell subsets simultaneously. For T cells, CD4^+^ and CD8^+^ fractions were assessed together with CD57, Ki67, and PD-1. NK cells were classified into three subsets by CD56/CD16 intensity: CD56hiCD16^−^ (regulatory), CD56d^im^CD16^+^ (cytotoxic), and CD56^−^CD16^+^ (atypical), with Ki67 and PD-1 co-assessed. B cells were classified into four subsets by IgD and CD27: IgD^+^ CD27^−^ (naive), IgD^+^CD27^+^ (marginal zone-like), IgD^−^CD27^+^ (switched memory), and IgD^−^CD27^−^ (double-negative/ABCs), with Ki67 included. The gating hierarchy started from CD45^+^ lymphocytes (by FSC/SSC), with CD3^+^ cells gated as T cells, CD3^−^CD19^+^ as B cells, and CD3^−^CD56^+^/CD16^+^ as NK cells. Representative gating strategies for both panels are provided in Supplementary Figure S1. Antibody details are provided in Table 2.

**TABLE 2:** Panel 1 antibody panel.

Channel	Fluorochrome	Antibody	Clone	Vendor
V1 (BV421)	Brilliant Violet 421	Ki-67	Ki-67	BioLegend
V2 (BV510)	Brilliant Violet 510	CD3	OKT3	BioLegend
V3 (BV605)	Brilliant Violet 605	IgD	IA6-2	BioLegend
V4 (BV711)	Brilliant Violet 711	CD57	QA17A04	BioLegend
B1 (FITC)	FITC	CD27	O323	BioLegend
Y2 (PE)	PE	CD19	HIB19	BioLegend
Y3 (PE/Dazzle 594)	PE/Dazzle™ 594	PD-1 (CD279)	EH12.2H7	BioLegend
Y4 (PE/Cy7)	PE/Cyanine7	CD56	5.1H11	BioLegend
R1 (PerCP-Cy5.5)	PerCP/Cyanine5.5	CD4	RPA-T4	BioLegend
R3 (APC)	APC	CD16	3G8	BioLegend
R4 (APC/Cy7)	APC/Cyanine7	CD8a	RPA-T8	BioLegend
R5 (AF700)	Alexa Fluor® 700	CD45	2D1	BioLegend

All antibodies from BioLegend.

Panel 2 was a 12-color dual-tube assay (n = 43) focused on T cell differentiation state. Tube 1 (T cell tube) combined CD45RA (FITC), CD62L (APC), CD28 (PE/Cy7), CD38 (BV605), HLA-DR (BV510), CD31 (PE), CD4 (PerCP-Cy5.5), CD8a (APC/Cy7), CD57 (BV711), Ki67 (BV421), PD-1 (PE/Dazzle 594), and CD45 (AF700). Within the CD4^+^ and CD8^+^ gates, CD45RA/CD62L allowed resolution of four subsets: naive (CD45RA^+^ CD62L^+^), central memory (TCM, CD45RA^−^CD62L^+^), effector memory (TEM, CD45RA^−^CD62L^−^), and TEMRA (CD45RA^+^ CD62L^−^); the TEMRA fraction was calculated as total CD45^RA+^ minus naive. Tube 2 (B/NK tube) used the same antibody configuration as Panel 1. At least 100,000 lymphocyte events were acquired per tube. Antibody details for both panels are provided in Tables 2 and 3.

**TABLE 3:** Panel 2 antibody panel (dual-tube).

Channel	Fluorochrome	Antibody	Clone	Vendor	Tube
Tube 1 (T cell tube, 12-color)					
V1 (BV421)	Brilliant Violet 421	Ki-67	Ki-67	BioLegend	1
V2 (BV510)	Brilliant Violet 510	HLA-DR	L243	BioLegend	1
V3 (BV605)	Brilliant Violet 605	CD38	HIT2	BioLegend	1
V4 (BV711)	Brilliant Violet 711	CD57	QA17A04	BioLegend	1
B1 (FITC)	FITC	CD45RA	HI100	BioLegend	1
Y2 (PE)	PE	CD31	WM59	BioLegend	1
Y3 (PE/Dazzle 594)	PE/Dazzle™ 594	PD-1	EH12.2H7	BioLegend	1
Y4 (PE/Cy7)	PE/Cyanine7	CD28	CD28.2	BioLegend	1
R1 (PerCP-Cy5.5)	PerCP/Cyanine5.5	CD4	RPA-T4	BioLegend	1
R3 (APC)	APC	CD62L	DREG-56	BioLegend	1
R4 (APC/Cy7)	APC/Cyanine7	CD8a	RPA-T8	BioLegend	1
R5 (AF700)	Alexa Fluor® 700	CD45	2D1	BioLegend	1
Tube 2 (B/NK tube, 12-color, same as panel 1)					
V1 (BV421)	Brilliant Violet 421	Ki-67	Ki-67	BioLegend	2
V2 (BV510)	Brilliant Violet 510	CD3	OKT3	BioLegend	2
V3 (BV605)	Brilliant Violet 605	IgD	IA6-2	BioLegend	2
V4 (BV711)	Brilliant Violet 711	CD57	QA17A04	BioLegend	2
B1 (FITC)	FITC	CD27	O323	BioLegend	2
Y2 (PE)	PE	CD19	HIB19	BioLegend	2
Y3 (PE/Dazzle 594)	PE/Dazzle™ 594	PD-1	EH12.2H7	BioLegend	2
Y4 (PE/Cy7)	PE/Cyanine7	CD56	5.1H11	BioLegend	2
R1 (PerCP-Cy5.5)	PerCP/Cyanine5.5	CD4	RPA-T4	BioLegend	2
R3 (APC)	APC	CD16	3G8	BioLegend	2
R4 (APC/Cy7)	APC/Cyanine7	CD8a	RPA-T8	BioLegend	2
R5 (AF700)	Alexa Fluor® 700	CD45	2D1	BioLegend	2

Tube 1, T cell tube; Tube 2, B/NK, cell tube. Shared markers: CD45-AF700, CD4-PerCP-Cy5.5, CD8a-APC/Cy7, PD-1-PE/Dazzle 594, Ki67-BV421, CD57-BV711. All antibodies from BioLegend.

### 2.4 Statistical analysis

Statistical analysis was performed in SPSS 26.0 and Python 3.10 (scipy, pandas). Continuous variables are reported as median (interquartile range, IQR). Age associations were assessed by Spearman rank correlation, reported as r and two-tailed p values. Participants were stratified into three age groups: young (20–44 years), middle-aged (45–64 years), and older (≥65 years); between-group comparisons used the Kruskal–Wallis test with Dunn *post hoc* testing and Bonferroni correction where indicated. Sex differences were tested by Mann-Whitney U test. Multivariate analysis employed partial Spearman correlations controlling for sex, supplemented by ordinary least squares (OLS) regression with standardized age and sex as covariates. A two-tailed p < 0.05 was considered statistically significant.

## 3 Results

### 3.1 Study population

The two cohorts together comprised 146 healthy adults with no participant overlap. Panel 1 covered ages 20–82 years (mean 45.3 ± 14.0), with 52 young (50.5%), 39 middle-aged (37.9%), and 12 older participants (11.7%); 83.5% were male. Panel 2 spanned ages 25–92 years (mean 63.6 ± 19.8), with 62.8% older participants and a somewhat higher proportion of women (32.6%). Detailed demographic data appear in Table 1.

### 3.2 T cell changes in panel 1 (n = 103)

The two strongest age correlations in Panel 1 were both CD57-related: CD4^+^CD57^+^ (*r* = +0.338, p < 0.001) and CD8^+^CD57^+^ (*r* = +0.308, p = 0.002) rose consistently with age, reflecting progressive accumulation of terminally differentiated T cells across both subsets (Figure 1).

**FIGURE 1:**
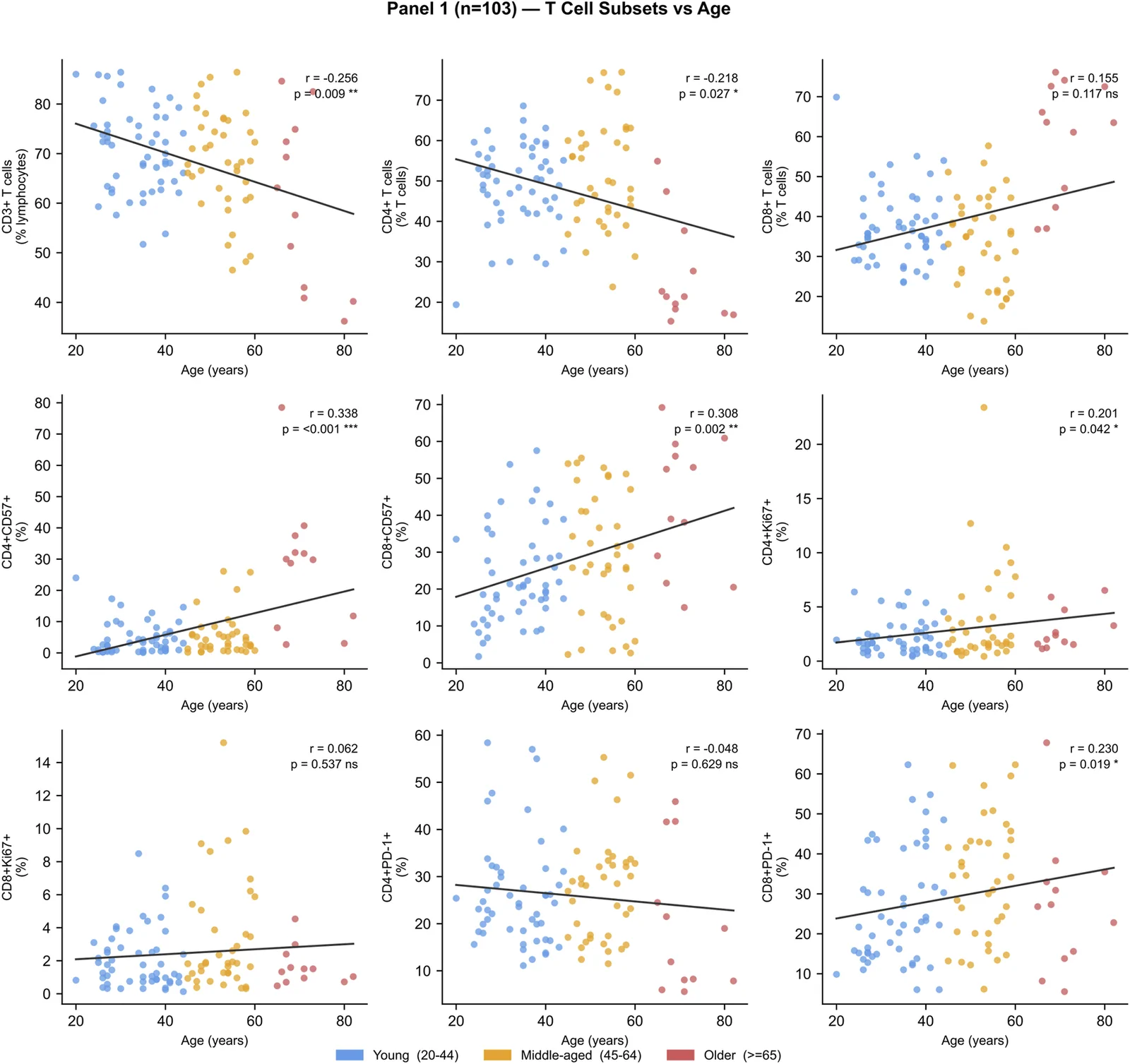
Scatter plots of Panel 1 T cell parameters *versus* age. Each panel shows age (years, x-axis) against the indicated T cell subset frequency (%). Blue circles, males; pink triangles, females. Dashed lines represent Spearman regression fits. Spearman r and p values are shown in each panel (*p < 0.05, **p < 0.01, ***p < 0.001, ns = not significant). Subpanels from left to right: total CD3^+^ T cells (% lymphocytes), CD4^+^ T cells (% T cells), CD8^+^ T cells (% T cells), CD4^+^CD57^+^, CD8^+^CD57^+^, CD4^+^Ki67^+^, CD8^+^Ki67^+^, CD4^+^PD-1^+^, CD8^+^PD-1^+^. n = 103.

Beyond CD57, the CD4^+^ fraction (as a proportion of T cells) declined with age (*r* = −0.218, p = 0.027), while the CD8^+^ proportion rose correspondingly (*r* = +0.155, p = 0.117), driving a downward shift in the CD4/CD8 ratio. Overall T cell frequency also declined (*r* = −0.256, p = 0.009). CD4^+^Ki67^+^ showed a modest but significant increase (*r* = +0.201, p = 0.042), consistent with compensatory homeostatic proliferation. CD4^+^PD-1^+^ did not reach significance within Panel 1 alone (p = 0.629); CD8^+^PD-1^+^ however showed a significant positive correlation (*r* = +0.230, p = 0.019). When all 146 participants were pooled for the shared markers, both CD4^+^PD-1^+^ (*r* = +0.291, p < 0.001) and CD8^+^PD-1^+^ (*r* = +0.367, p < 0.001) became significantly positive, suggesting that statistical power rather than a true null effect explains the Panel 1 CD4^+^PD-1^+^ finding (see Table 4).

**TABLE 4:** Spearman correlations with age for Panel 1 parameters (n = 103 or n = 146).

Parameter	n	Spearman r	p value	Significance	Trend
T cell subsets					
CD3^+^ T cells (% lymphocytes)	103	−0.256	0.009	**	↓
CD4^+^ T cells (% T cells)	146	−0.362	<0.001	***	↓
CD8^+^ T cells (% T cells)	146	−0.153	0.065	ns	—
CD4^+^CD57^+^ T cells (%)	146	+0.421	<0.001	***	↑
CD8^+^CD57^+^ T cells (%)	146	+0.338	<0.001	***	↑
CD4^+^Ki67^+^ T cells (%)	146	+0.008	0.921	ns	—
CD8^+^Ki67^+^ T cells (%)	146	−0.001	0.994	ns	—
CD4^+^PD-1^+^ T cells (%)	146	+0.291	<0.001	***	↑
CD8^+^PD-1^+^ T cells (%)	146	+0.367	<0.001	***	↑
NK cell subsets					
NK cells (% lymphocytes)	146	+0.312	<0.001	***	↑
CD56hiCD16^−^ NK (% NK cells)	146	−0.335	<0.001	***	↓
CD56^dim^CD16^+^ NK (% NK cells)	146	−0.047	0.573	ns	—
CD56^−^CD16^+^ NK (% NK cells)	146	+0.133	0.109	ns	—
NK PD-1^+^ (%)	146	+0.318	<0.001	***	↑
NK Ki67^+^ (%)	146	+0.010	0.903	ns	—
B cell subsets					
B cells (% lymphocytes)	146	−0.133	0.110	ns	—
IgD^+^ CD27^−^ naive B cells (%)	146	+0.155	0.061	ns	—
IgD^+^CD27^+^ marginal zone-like B cells (%)	103	−0.257	0.009	**	↓
IgD^−^CD27^+^ switched memory B cells (%)	146	−0.169	0.042	*	↓
IgD^−^CD27^−^ double-negative B cells/ABCs (%)	146	−0.001	0.991	ns	—
B Ki67^+^ (%)	146	−0.152	0.068	ns	—

ABCs, age-associated B cells; * p < 0.05; ** p < 0.01; *** p < 0.001; ns, not significant.

CD3^+^ T cells (% lymphocytes) and IgD^+^CD27^+^ marginal zone-like B cells were available only in Panel 1 (n = 103); all other parameters were analyzed in the full 146-participant dataset (Panel 1 n = 103 + Panel 2 n = 43) for shared baseline markers.

### 3.3 T cell changes in panel 2

#### 3.3.1 CD8^+^ T cells

The Panel 2 data told a particularly clear story for CD8^+^ T cells (Figure 2). TEMRA accumulation (*r* = +0.532, p < 0.001), CD28 loss (*r* = −0.552, p < 0.001), and naive cell depletion (*r* = −0.534, p < 0.001) formed a tight triad of age-associated changes, all reaching p < 0.001 (Table 5). Together they paint a picture of progressive, co-stimulation-independent terminal differentiation that dominates the CD8^+^ compartment in older individuals. Activation and proliferation markers reinforced this: CD8^+^HLA-DR^+^ (*r* = +0.370, p = 0.015), CD8^+^CD38^+^HLA-DR^+^ (*r* = +0.363, p = 0.017), and CD8^+^Ki67^+^ (*r* = +0.375, p = 0.013) all rose with age, indicating that despite their differentiated phenotype, CD8^+^ T cells in older adults remain in a state of chronic activation. Total CD8^+^ memory frequency (CD45RA^−^) showed no net change, nor did CD8^+^CD57^+^ or CD8^+^PD-1^+^ within this smaller cohort.

**FIGURE 2:**
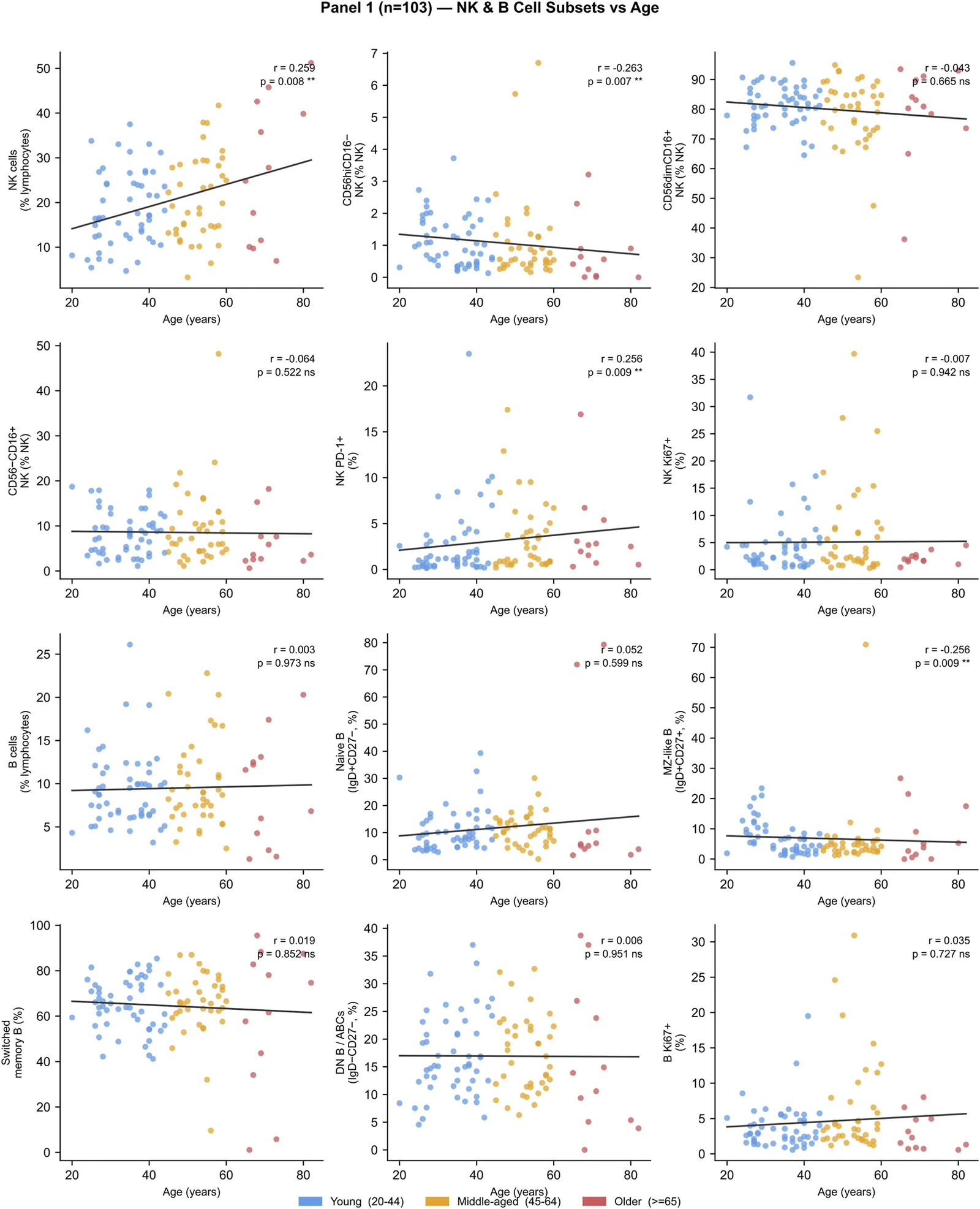
Scatter plots of Panel 1 NK and B cell parameters *versus* age. Format as in Figure 1. Subpanels: total NK cells (% lymphocytes), CD56hiCD16− NK (% NK cells), CD56dimCD16^+^ NK (% NK cells), NK Ki67^+^, NK PD-1^+^, total B cells (% lymphocytes), B Ki67^+^, IgD ^+^ CD27^−^ naive B cells (% B cells), IgD^−^CD27^−^ double-negative B cells/ABCs (% B cells), CD56-CD16^+^NK, IgD^+^CD27^+^ marginal zone-like B cells, and IgD-CD27^+^ switched memory B cells. n = 103.

**TABLE 5:** Spearman correlations with age for Panel 2 T cell parameters (n = 43).

Parameter	n	Spearman r (95% CI)	p value	Significance	Trend
CD8^+^ T cell deep phenotype					
CD8^+^ naive (CD45RA^+^ CD62L^+^, %)	43	−0.534 [−0.719, −0.278]	<0.001	***	↓
CD8^+^ TEMRA (CD45RA^+^ CD62L^−^, %)	43	+0.532 [0.276, 0.718]	<0.001	***	↑
CD8^+^ memory (CD45RA^−^, %)	43	+0.065 [−0.240, 0.358]	0.681	ns	—
CD8^+^CD28^+^ (%)	43	−0.552 [−0.731, −0.301]	<0.001	***	↓
CD8^+^CD31^+^ (%)	43	−0.232 [−0.498, 0.073]	0.134	ns	—
CD8^+^CD38^+^ (%)	43	+0.012 [−0.289, 0.312]	0.937	ns	—
CD8^+^HLA-DR^+^ (%)	43	+0.370 [0.078, 0.603]	0.015	*	↑
CD8^+^CD38^+^HLA-DR^+^ (%)	43	+0.363 [0.070, 0.598]	0.017	*	↑
CD8^+^Ki67^+^ (%)	43	+0.375 [0.085, 0.607]	0.013	*	↑
CD4^+^ T cell deep phenotype					
CD4^+^ naive (CD45RA^+^ CD62L^+^, %)	43	−0.303 [−0.553, −0.003]	0.048	*	↓
CD4^+^ TEMRA (CD45RA^+^ CD62L−, %)	43	−0.006 [−0.305, 0.295]	0.971	ns	—
CD4^+^ memory (CD45RA−, %)	43	+0.276 [−0.026, 0.532]	0.073	ns	—
CD4^+^CD28^+^ (%)	43	+0.051 [−0.253, 0.346]	0.743	ns	—
CD4^+^CD31^+^ (%)	43	−0.361 [−0.597, −0.068]	0.017	*	↓
CD4^+^CD38^+^ (%)	43	−0.239 [−0.503, 0.066]	0.123	ns	—
CD4^+^HLA-DR^+^ (%)	43	−0.085 [−0.375, 0.221]	0.589	ns	—
CD4^+^CD38^+^HLA-DR^+^ (%)	43	−0.260 [−0.520, 0.043]	0.092	ns	—

TEMRA, terminally differentiated effector memory T cells re-expressing CD45RA; RTE, recent thymic emigrants. * p < 0.05; ** p < 0.01; *** p < 0.001; ns, not significant.

#### 3.3.2 CD4^+^ T cells

Two significant age-related changes were identified in the CD4^+^ compartment (Figure 3). First, CD4^+^CD31^+^ cells declined with age (*r* = −0.361, p = 0.017); as a marker of recent thymic emigrants, this drop is consistent with thymic involution progressively curtailing new naive T cell output in older individuals. Second, CD4^+^ naive cells (CD45^RA+^ CD62L^+^) also showed a significant age-related decline (*r* = −0.303, p = 0.048), reinforcing the picture of progressive naive T cell attrition. None of the other CD4^+^ parameters—including TEMRA, memory, CD28^+^, CD38^+^, HLA-DR^+^, CD57^+^, Ki67^+^, or PD-1^+^ fractions—showed a statistically significant age relationship in this cohort (Figures 4, 5).

**FIGURE 3:**
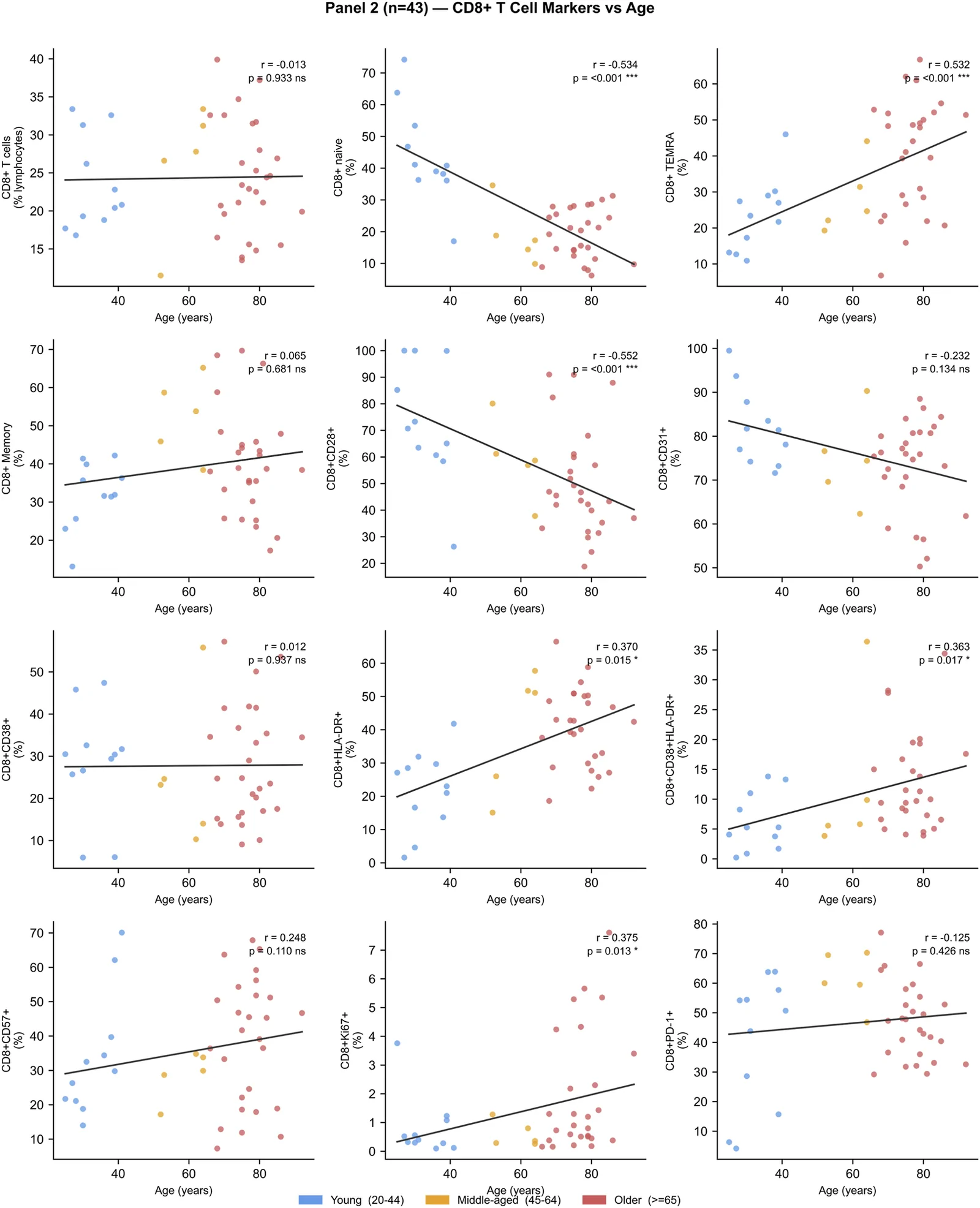
Scatter plots of Panel 2 CD8^+^ T cell parameters *versus* age. Format as in Figure 1. Subpanels: CD8^+^CD28^+^, CD8^+^ naive (CD45^RA+^ CD62L^+^), CD8^+^ TEMRA (CD45RA^+^ CD62L^−^), CD8^+^ memory (CD45RA^−^), CD8^+^HLA-DR^+^, CD8^+^CD38^+^HLA-DR^+^, CD8^+^CD57^+^, CD8^+^Ki67^+^, CD8^+^PD-1^+^, CD8^+^CD31^+^ (RTE), CD8^+^CD38^+^, total CD8^+^ T cells (% lymphocytes). n = 43.

**FIGURE 4:**
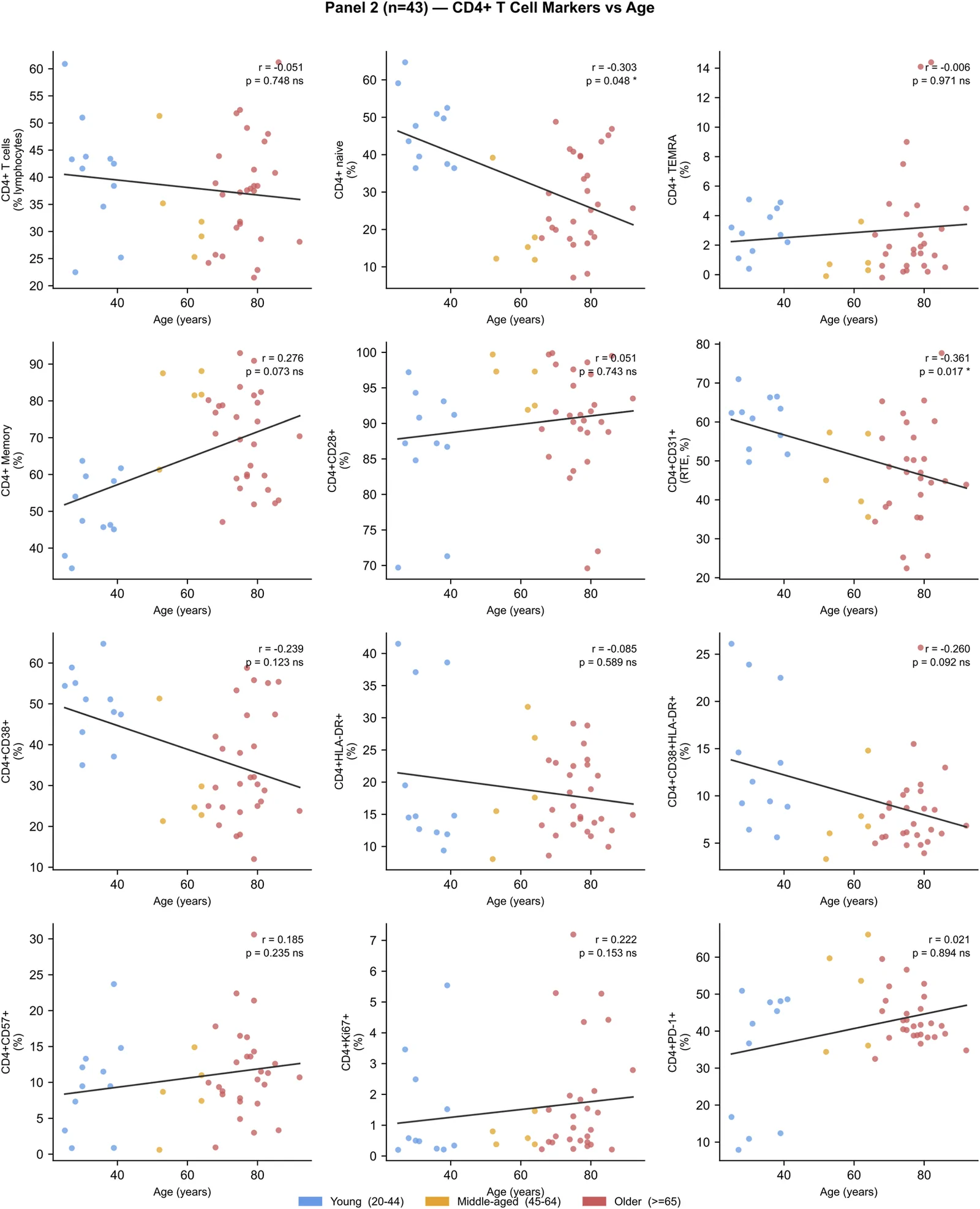
Scatter plots of Panel 2 CD4^+^ T cell parameters *versus* age. Format as in Figure 1. Subpanels: CD4^+^CD28^+^, CD4^+^ naive (CD45RA^+^ CD62L^+^), CD4^+^ TEMRA (CD45RA^+^ CD62L^−^), CD4^+^ memory (CD45RA^−^), CD4^+^HLA-DR^+^, CD4^+^CD38^+^HLA-DR^+^, CD4^+^CD57^+^, CD4^+^Ki67^+^, CD4^+^PD-1^+^, CD4^+^CD31^+^ (RTE), CD4^+^CD38^+^, total CD4^+^ T cells (% lymphocytes). n = 43.

**FIGURE 5:**
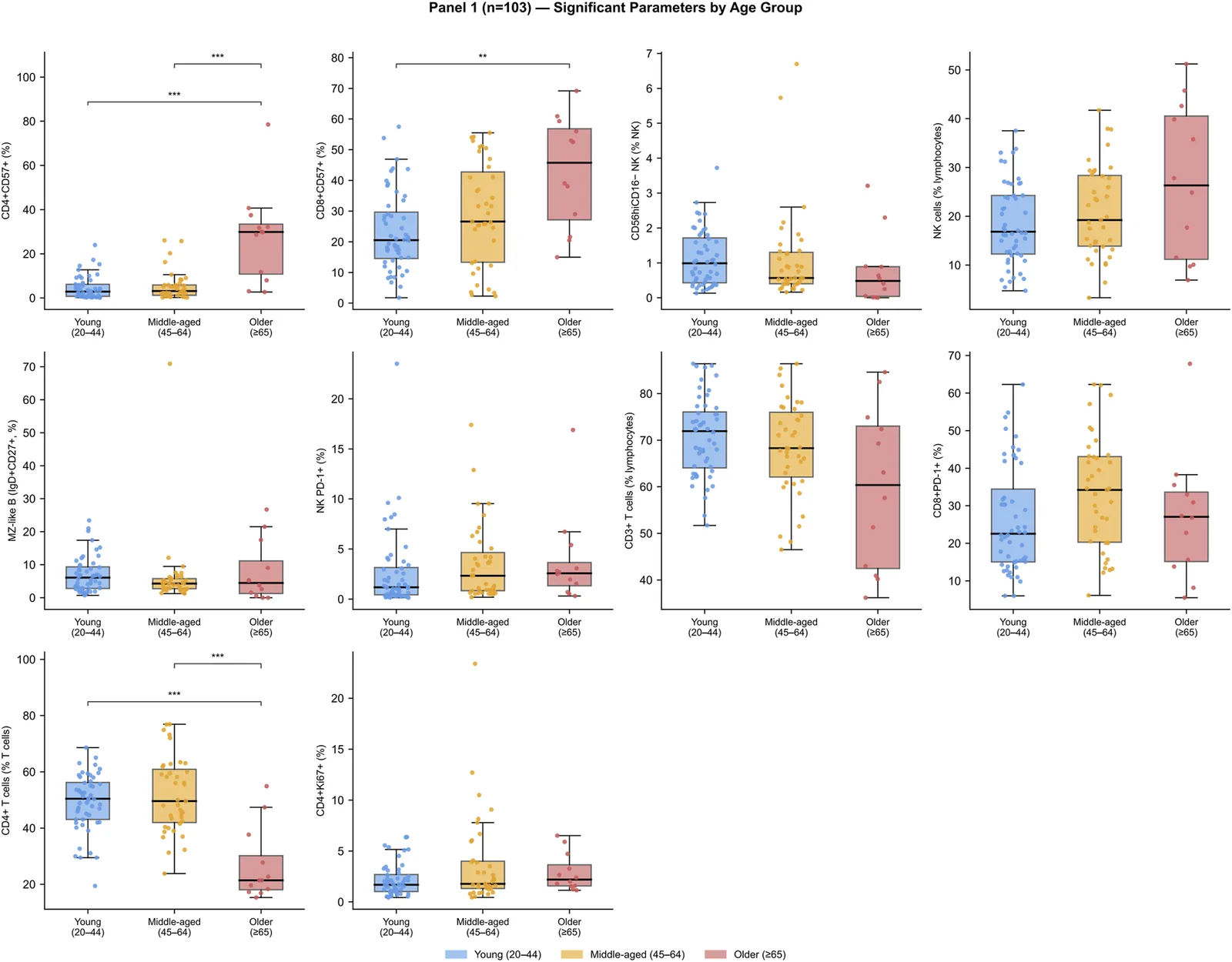
Box plots of key Panel 1 parameters stratified by age group. Participants divided into young (20–44 years, n = 52), middle-aged (45–64 years, n = 39), and older (≥65 years, n = 12) groups. Boxes show median and IQR; whiskers extend to 1.5×IQR; open circles are outliers. blue, young; orange, middle-aged; red, older. Kruskal–Wallis p values are shown. Subpanels: total T cells, CD4^+^ T cells, CD8^+^ T cells, CD4^+^CD57^+^, CD8^+^CD57^+^, NK cells, CD56hiCD16^−^ NK, NK PD-1^+^, B cells, CD4^+^Ki67^+^.

### 3.4 NK cell changes

Panel 1 (n = 103) showed a clear age-associated rise in total NK cell frequency (*r* = +0.259, p = 0.008) (Figure 6). Within that expanding NK pool, however, the CD56hiCD16^−^ regulatory subset contracted (*r* = −0.263, p = 0.007), while CD56dimCD16^+^ and CD56^−^CD16^+^ showed no significant change. NK PD-1 expression also rose with age (*r* = +0.256, p = 0.009). NK Ki67^+^ was unchanged (p = 0.942).

**FIGURE 6:**
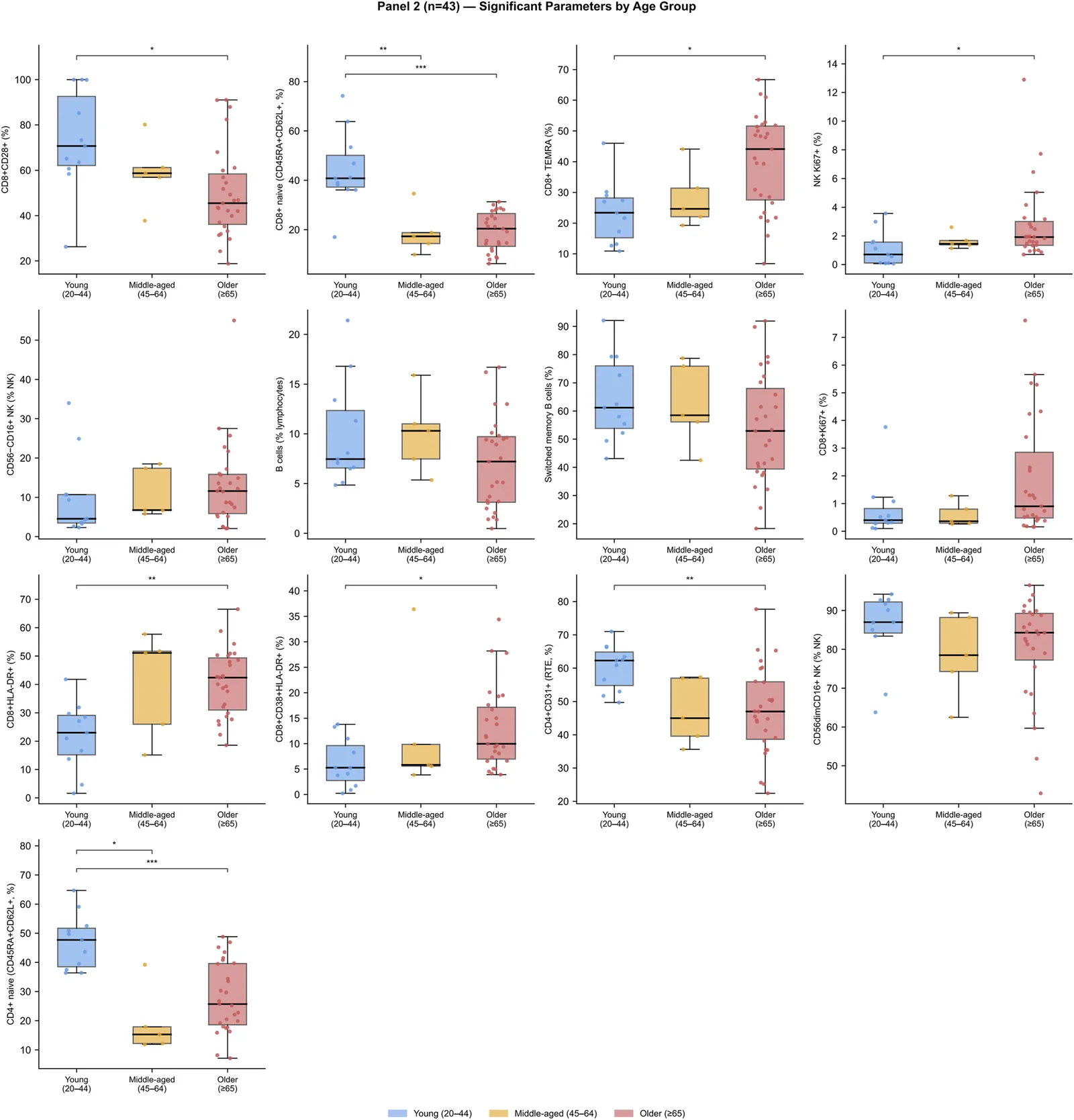
Box plots of key Panel 2 parameters stratified by age group. Groups as in Figure 5 (young n = 11, middle-aged n = 5, older n = 27). Subpanels: CD4^+^CD28^+^; CD8^+^ naive; CD8^+^ TEMRA; NK Ki67^+^; CD8^+^CD31^+^ (% NK); B cells (% lymphocytes); switched memory B cells; CD4^+^Ki67^+^; CD8^+^HLA-DR^+^; CD8^+^CD38^+^HLA-DR^+^; CD4^+^CD31^+^ (RTE); CD56dimCD16^+^ NK (% NK); CD4^+^ naive (CD45RA^+^CD62L^+^).

In the combined dataset (n = 146), segmented Spearman analysis with age 65 as the breakpoint showed that CD56^−^CD16^+^ NK cells were non-significant overall (*r* = +0.133, p = 0.109) but reached significance in participants aged ≥65 years (n = 39, *r* = +0.482, 95% CI [0.196, 0.692], p = 0.002), compared with non-significance in those under 65 (n = 107, *r* = +0.123, p = 0.206). The formal slope-change test did not reach significance (p = 0.094), so this pattern is best described as a late-life tendency requiring replication rather than a confirmed threshold effect. For CD56^dim^CD16^+^ NK cells, neither age segment showed a significant association in the combined analysis. In Panel 2, NK Ki67^+^ showed a significant positive correlation (*r* = +0.420, 95% CI [0.137, 0.640], p = 0.005). Segmented regression results are shown in Figure 7.

**FIGURE 7:**
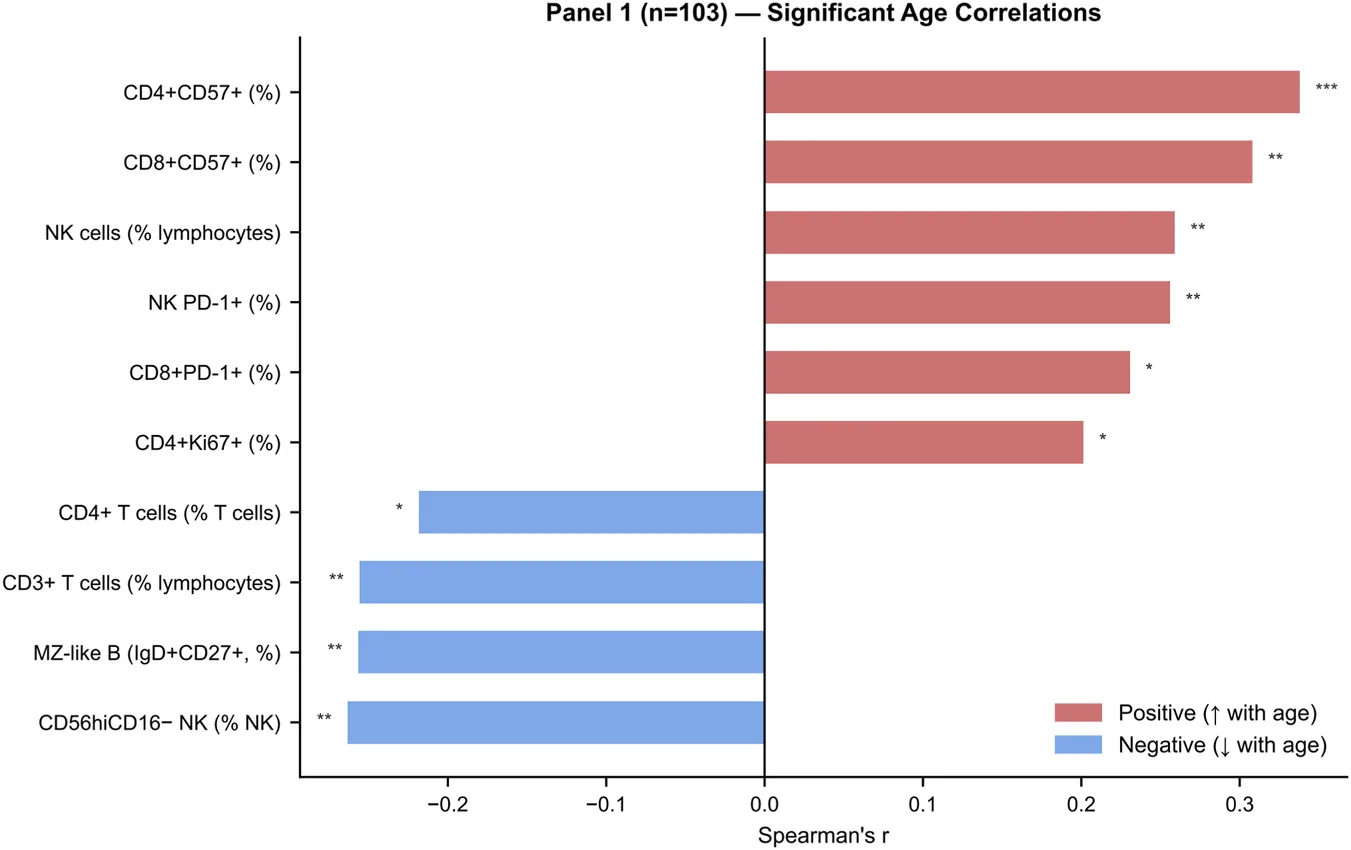
Summary bar chart of Spearman r values for all Panel 1 parameters *versus* age. Horizontal bars show Spearman r for all 21 Panel 1 markers, ranked from highest to lowest. Blue, positive correlations; red, negative correlations. Significance levels marked on the right (*p < 0.05, **p < 0.01, ***p < 0.001). n = 103.

### 3.5 B Cell changes

Total B cell frequency was stable across the Panel 1 age range (*r* = +0.003, p = 0.973) (Figures 8, 9). Among the four IgD/CD27 subsets, IgD^+^CD27^+^ marginal zone-like B cells showed a significant age-related decline in Panel 1 (*r* = −0.257, p = 0.009). None of the other three subsets—naive, switched memory, or double-negative/ABCs—reached significance in Panel 1, nor did B Ki67^+^.

**FIGURE 8:**
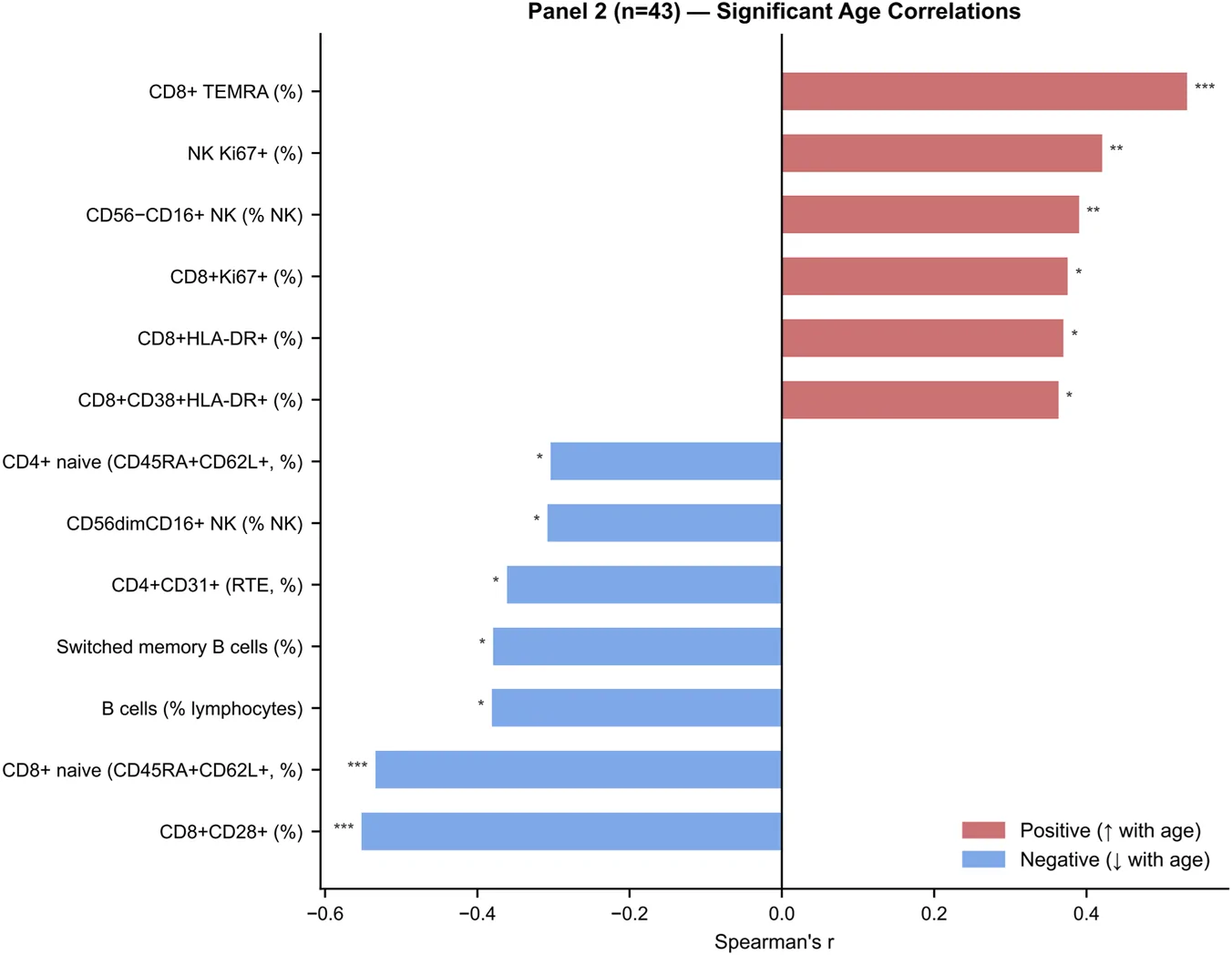
Summary bar chart of Spearman r values for all Panel 2 parameters *versus* age. As in Figure 7, for all Panel 2 markers. n = 43.

**FIGURE 9:**
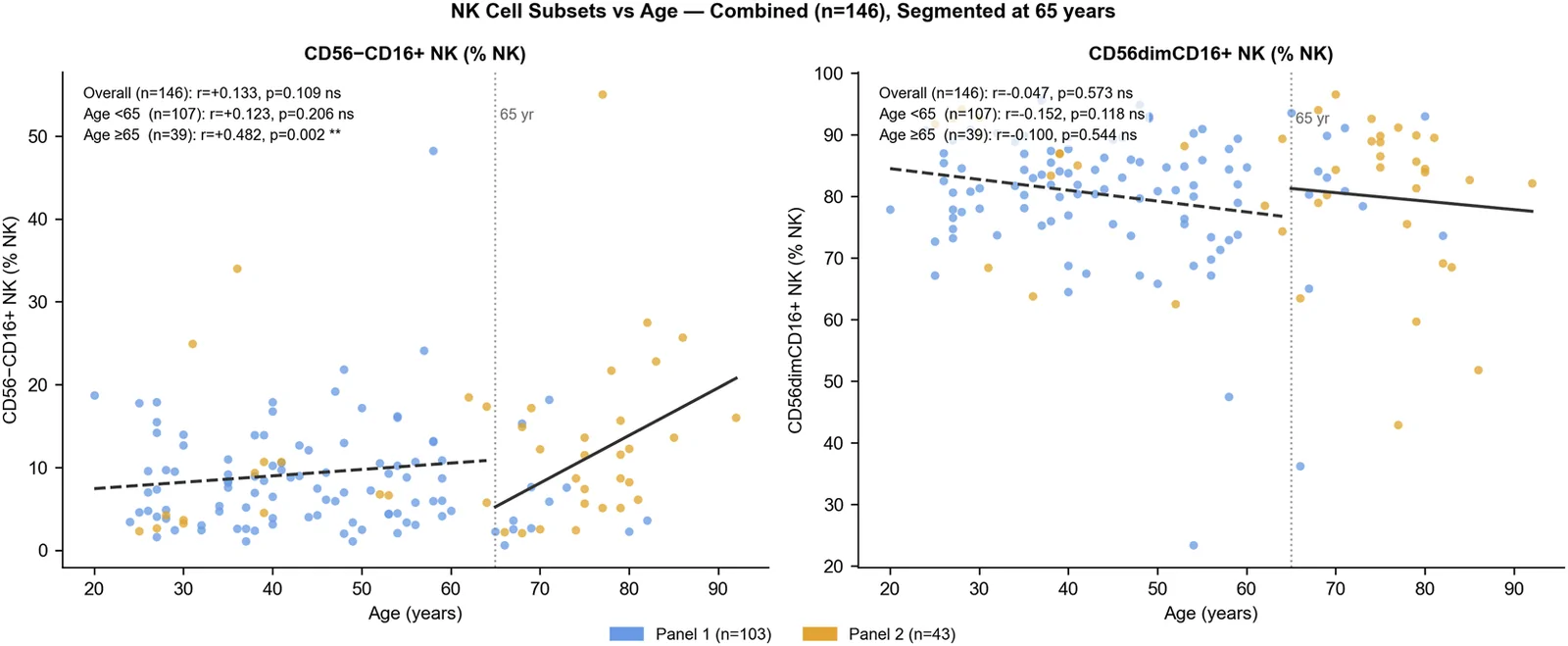
Segmented Spearman correlation analysis for CD56^−^CD16^+^ and CD56dimCD16^+^ NK cell subsets in the combined dataset (n = 146), with breakpoint at age 65 years. Each subpanel shows age (years, x-axis) against NK subset frequency (% NK, y-axis). Blue circles, Panel 1 (n = 103); orange circles, Panel 2 (n = 43). Dashed line, regression fit for age <65 years; solid line, regression fit for age ≥65 years. Overall Spearman r and segmented Spearman r with p values are shown in each subpanel.

Panel 2 presented a more pronounced picture at older ages: total B cell frequency declined significantly (*r* = −0.381, p = 0.012) and switched memory B cells contracted (*r* = −0.379, p = 0.012). That these changes appeared in an older-enriched cohort (median age 70, maximum 92) but not in the broader Panel 1 sample suggests that substantial B cell and memory contraction may be a relatively late phenomenon, becoming apparent mainly in the oldest age ranges, consistent with functional data from Frasca and colleagues (Frasca and Blomberg, 2014).

### 3.6 Sex effects and multivariate analysis

In Panel 1 (n = 103), five parameters differed significantly by sex (Mann-Whitney U, p < 0.05). Women had lower CD4^+^ fractions (p = 0.011) and higher CD8^+^ fractions (p = 0.003) than men, suggesting a modestly more differentiated T cell phenotype in women at a given age. Men had higher CD8^+^PD-1^+^ (p = 0.040). B cell sex differences were also apparent: men had higher naive B (p = 0.028) and ABC proportions (p < 0.001) than women. In Panel 2, five parameters differed by sex: women had higher total CD8^+^ (p = 0.010), CD4^+^CD31^+^ (p = 0.015), and CD8^+^CD31^+^ (p<0.001) fractions, while men had higher NK frequency (p = 0.023) and CD8^+^ memory fractions (p = 0.013).

Multivariate analysis (partial Spearman correlations controlling for sex, plus OLS regression) confirmed that the vast majority of age-related findings were independent of sex. In Panel 1, all ten significant univariate associations held after sex adjustment, including CD4^+^CD57^+^ (partial *r* = +0.337, p < 0.001), CD8^+^CD57^+^ (partial *r* = +0.315, p = 0.001), CD4 T% (partial *r* = −0.217, p = 0.028), NK% (partial *r* = +0.265, p = 0.007), CD56^hi^CD16^−^ NK% (partial *r* = −0.267, p = 0.006), total T% (partial *r* = −0.258, p = 0.009), NK PD-1^+^ (partial *r* = +0.266, p = 0.007), CD8^+^PD-1^+^ (partial *r* = +0.243, p = 0.013), CD4^+^Ki67^+^ (partial *r* = +0.202, p = 0.041), and MZ-like B (partial *r* = −0.258, p = 0.009). In Panel 2, all three core CD8^+^ metrics—TEMRA (partial *r* = +0.537), naive (partial *r* = −0.547), and CD28^+^ (partial *r* = −0.551) — remained strongly significant (all p < 0.001) after controlling for sex.

### 3.7 Immunological Composite Score

To integrate the multidimensional age signal into a single summary measure, we constructed an Immunological Composite Score (ICS) from nine parameters that showed consistent, sex-independent age associations across both panels: CD4^+^CD57^+^, CD8^+^CD57^+^, CD4^+^PD-1^+^, CD8^+^PD-1^+^, NK%, and NK PD-1^+^ (each contributing positively) and CD4% T cells, CD56hiCD16^−^ NK%, and switched memory B% (each contributing negatively). Each parameter was z-scored and combined with directional signs aligned to the age correlation direction.

Across all 146 participants, the ICS correlated strongly with chronological age (Spearman *r* = +0.575, p < 0.001). Kruskal–Wallis testing demonstrated highly significant group separation (H = 49.78, p < 0.001). Dunn *post hoc* comparisons showed that the older group (≥65 years; median ICS +4.85, IQR +2.45 to +6.53) differed significantly from both the young group (20–44 years; median −2.39, IQR −5.27 to +0.34; p < 0.001) and the middle-aged group (45–64 years; median −0.78, IQR −2.50 to +1.82; p < 0.001), while the young and middle-aged groups did not differ significantly from each other (p = 0.486). The ICS is presented as a proof-of-concept instrument; external prospective validation is required before clinical application (Figure 10).

**FIGURE 10:**
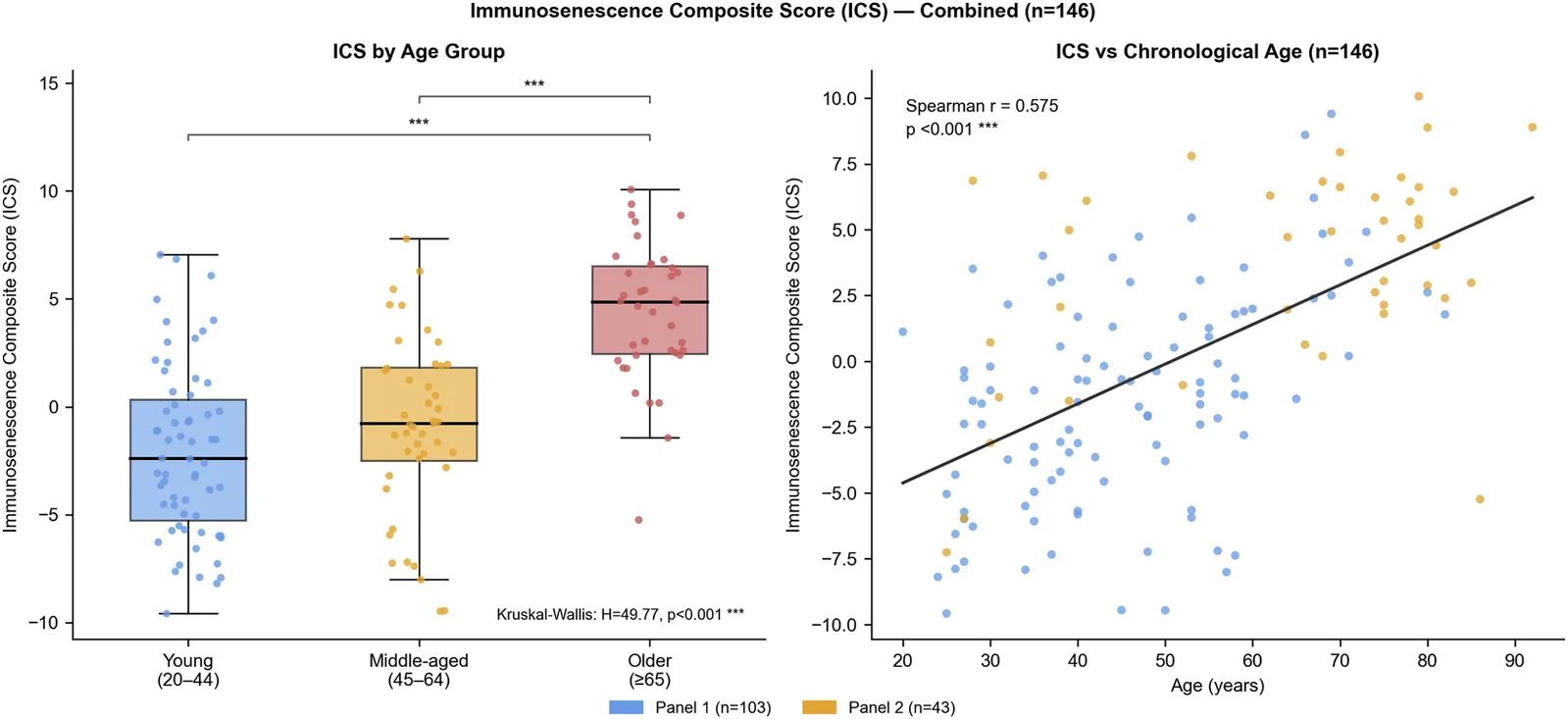
Immunological Composite Score (ICS) across age groups and in relation to chronological age. Left, box plot of ICS stratified by age group (young 20–44 years, n = 63; middle-aged 45–64 years, n = 44; older ≥65 years, n = 39) in the combined dataset (n = 146); boxes show median and IQR, whiskers 1.5 × IQR, individual data points overlaid; blue, Panel 1 (n = 103); orange, Panel 2 (n = 43); Kruskal–Wallis H and Dunn *post hoc* significance are shown (***p < 0.001). Right, scatter plot of ICS *versus* chronological age (n = 146); solid line, regression fit; Spearman r and p value shown.

### 3.8 Cross-lineage senescence correlations

To assess whether age-associated immune changes were coordinated across lineages within the same individuals, we performed pairwise Spearman correlations between senescence markers from different compartments. In Panel 1 (n = 103), the strongest cross-lineage association was between CD4^+^CD57^+^ and CD8^+^CD57^+^ (*r* = +0.469, p < 0.001), indicating that terminal T cell differentiation proceeds in parallel across both subsets. CD4^+^CD57^+^ was also inversely correlated with the CD56^hi^CD16^−^ regulatory NK subset (*r* = −0.320, p = 0.001). Positive associations were observed between CD8^+^PD-1^+^ and DN B/ABCs (*r* = +0.294, p = 0.003), between CD8^+^CD57^+^ and NK PD-1^+^ (*r* = +0.288, p = 0.003), and between NK PD-1^+^ and DN B/ABCs (*r* = +0.255, p = 0.009). In Panel 2 (n = 43), CD8^+^ TEMRA accumulation was tightly coupled with CD8^+^CD28 loss (*r* = −0.775, p < 0.001) and with CD8^+^CD57^+^ accumulation (*r* = +0.557, p < 0.001), and NK PD-1^+^ correlated positively with CD56^−^CD16^+^ NK expansion (*r* = +0.327, p = 0.032). Together, these findings suggest that immune aging is partially coordinated across T cell, NK cell, and B cell compartments within individuals (Figure 11; Supplementary Tables S5 and S6).

**FIGURE 11:**
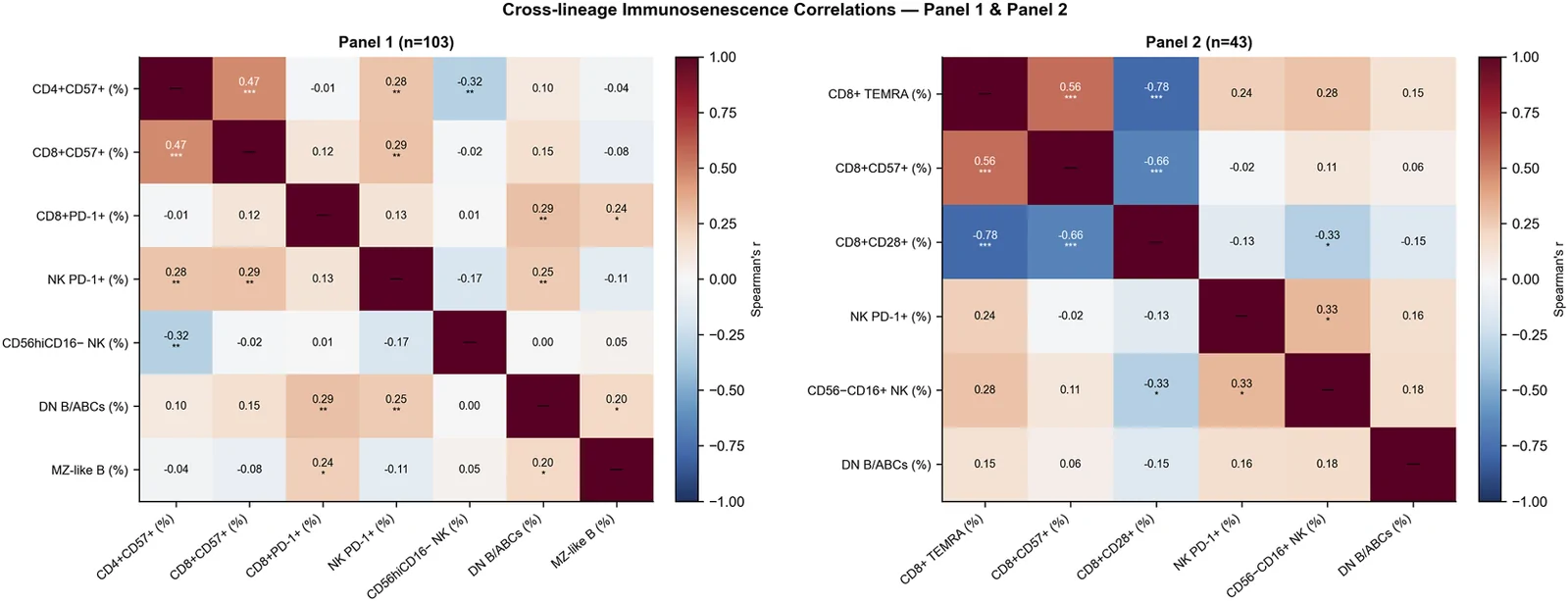
Cross-lineage immunosenescence correlation heatmaps. Left, Panel 1 (n = 103): pairwise Spearman correlations among CD4^+^CD57^+^, CD8^+^CD57^+^, CD8^+^PD-1^+^, NK PD-1^+^, CD56hiCD16^−^ NK%, DN B/ABCs%, and MZ-like B%. Right, Panel 2 (n = 43): pairwise Spearman correlations among CD8^+^ TEMRA, CD8^+^CD57^+^, CD8^+^CD28^+^, NK PD-1^+^, CD56^−^CD16^+^ NK%, and DN B/ABCs%. Color scale indicates Spearman r (red, positive; blue, negative). Significance annotated (*p < 0.05, **p < 0.01, ***p < 0.001). Diagonal cells masked.

## 4 Discussion

This study characterizes age-associated changes in peripheral blood NK cell subsets, B cell subsets, and T cell surface markers across a broad age spectrum (20–92 years) in 146 healthy Chinese adults, providing a multiparameter immunophenotyping reference dataset for this population.

The T cell data are arguably the most informative. In Panel 2, the shift of CD8^+^ T cells toward TEMRA (*r* = +0.532) was the single strongest age correlation we observed, occurring in concert with CD28 loss (*r* = −0.552) and naive cell depletion (*r* = −0.534). The three changes form a coherent triad—the hallmarks of replicative senescence in the CD8^+^ compartment—consistent with established features of T cell aging and with the epigenetic senescence signature of TEMRA cells documented in population-level studies (Fülöp et al., 2013; Salumets et al., 2022; Goronzy and Weyand, 2013). Layered onto this, both CD4^+^CD57^+^ and CD8^+^CD57^+^ rose significantly in Panel 1, reinforcing terminal differentiation as a dominant and lineage-wide feature of immune aging in this cohort. The concurrent decline of CD4^+^ naive cells (*r* = −0.303) and CD4^+^CD31^+^ RTE-like cells (*r* = −0.361) points to a parallel process: progressive thymic involution curtailing new naive T cell output, as anticipated from the known age-related loss of thymic function (Ferrando-Martínez et al., 2011). When the full 146-participant sample was analyzed for shared markers, both CD4^+^PD-1^+^ and CD8^+^PD-1^+^ showed robust age correlations (*r* = +0.291 and +0.367 respectively), consistent with the view that exhaustion-like checkpoint upregulation accompanies terminal differentiation as a co-occurring feature of immune aging in this population (Chang et al., 2024).

The NK cell findings are more nuanced. The overall rise in NK frequency with age, while the CD56hiCD16^−^ regulatory subset contracts, is consistent with the shift toward cytotoxic NK predominance described in other populations (Hazeldine and Lord, 2015). In the combined dataset (n = 146), segmented analysis revealed that CD56^−^CD16^+^ NK cells showed no significant overall age correlation but became significantly positively associated with age in participants aged ≥65 years (*r* = +0.482, 95% CI [0.196, 0.692], p = 0.002), compared with a non-significant association in those under 65 (*r* = +0.123, p = 0.206). The formal slope-change test did not reach significance (p = 0.094), so this pattern is best interpreted as a late-life tendency requiring replication in larger cohorts. This atypical subset was first characterized in the context of chronic HIV infection (Solana et al., 2012), and its selective expansion in the oldest participants likely reflects decades of cumulative antigenic exposure rather than frank immunodeficiency. The age-related rise in NK PD-1 expression—which emerged clearly in Panel 1 (*r* = +0.256) — is a less commonly reported observation and suggests that NK cells, like T cells, may undergo progressive phenotypic exhaustion with age. The mechanisms underlying this rise remain to be established, but likely include chronic low-grade inflammation (inflammaging) upregulating inhibitory receptors, cumulative stimulation by latent herpesviruses such as CMV driving an exhaustion-like NK phenotype, and replicative exhaustion following decades of immune activity. PD-1-expressing NK cells exhibit reduced cytotoxic capacity and impaired cytokine production; accordingly, age-related accumulation of PD-1^+^ NK cells may contribute to the diminished antitumor NK surveillance reported in older adults. Our NK subset findings are broadly consistent with observations from European cohorts (Le Garff-Tavernier et al., 2010), suggesting that the key features of NK cell aging may be conserved across populations.

B cell aging in this cohort followed a somewhat different timeline. In Panel 1, total B cell frequency was stable and the IgD/CD27 subsets were mostly unchanged, with only IgD^+^CD27^+^ marginal zone-like B cells showing a significant decline (*r* = −0.257). This subset represents antigen-experienced but unswitched B cells, and its contraction may reflect reduced T-independent responses with aging. The more dramatic changes—total B cell contraction and switched memory decline—emerged in Panel 2, in the oldest participants. Taken together, these observations suggest that B cell aging may be a relatively late-stage process, with meaningful contraction of the humoral memory compartment concentrated in the seventh decade and beyond, consistent with functional data from Frasca and colleagues (Frasca and Blomberg, 2014).

Sex was a meaningful source of variation throughout. Women in our cohort tended to have higher CD8^+^ fractions and lower CD4/CD8 ratios than men of the same age, a pattern in line with well-established sex differences in immune aging (Klein and Flanagan, 2016). Both panels showed higher CD31^+^ fractions in women, consistent with greater thymic output or slower naive T cell attrition in women. Men had higher NK frequency in Panel 2. Critically, however, multivariate analysis showed that sex did not explain the primary age effects; all ten significant age correlations in Panel 1 survived adjustment for sex, and the three core CD8^+^ markers (TEMRA, naive, CD28^+^) remained strongly significant regardless. The male-heavy composition of Panel 1 (83.5%) does introduce some interpretive caution, and we would encourage sex-stratified reporting when these data are used as clinical reference ranges. Specifically, the reference values reported here should be applied with caution to female adults, and sex-stratified reference intervals should be established in future studies with more balanced cohort recruitment.

This dataset sits alongside existing reference work from the same institution (Qin et al., 2016; Chang et al., 2024), which established total lymphocyte subset counts and described age trends for T cell markers including PD-1, Ki67, and CD31. The present data add NK cell three-subset classification, IgD/CD27 B cell four-subset classification, CD57 quantification, and TEMRA profiling—elements that together enable a more complete picture of peripheral immune aging in the Chinese adult population.

The ICS introduced in this study demonstrates that the multidimensional age signal captured by a routine 12-color clinical flow cytometry panel can be condensed into a single interpretable composite index. This approach requires no additional reagents or instrumentation beyond what is already used for standard lymphocyte subset analysis, making it practically accessible in clinical settings where multiparameter immunophenotyping is routinely performed. The ICS showed strong age correlation (*r* = +0.575) and clear separation between young and older groups, consistent with an acceleration of immune aging in the sixth decade and beyond. We emphasize, however, that the ICS was derived from and validated in the same dataset, introducing circularity that limits its current clinical utility. External prospective validation in an independent cohort—ideally one that includes longitudinal follow-up and clinical endpoints such as infection susceptibility or vaccine immunogenicity—is required before the ICS can be used as a clinical instrument.

Several limitations should be acknowledged. Panel 2 was small (n = 43) and skewed toward older participants, which improves sensitivity for age-related signals but limits generalizability of some findings. CMV serostatus was not collected, which is an acknowledged limitation. However, CMV is best understood as a mediating variable in the age→immune remodeling pathway rather than a simple external confounder. In Chinese adults, CMV seroprevalence exceeds 90% by the sixth decade (Noppert et al., 2024), meaning that latent CMV infection is itself an age-dependent biological reality rather than an independent exposure that can be cleanly removed. Evidence from both serological and epigenetic studies indicates that CMV-driven immune remodeling operates through the same senescence pathways activated by intrinsic aging (Salumets et al., 2022; Pawelec et al., 2012; Rodríguez and Parra-López, 2024; Sharpe et al., 2022), and the two processes are deeply intertwined in a seropositive population. For a reference dataset intended to reflect the immune phenotype as it actually exists in healthy Chinese adults, characterizing the landscape inclusive of CMV-associated remodeling is therefore appropriate. That said, future studies incorporating CMV serostatus data would allow stratified analyses and more precise attribution of individual immune changes. The cross-sectional design also means we cannot formally distinguish age effects from cohort effects. Notably, however, all participants underwent systematic health screening and none showed clinical signs of accelerated aging or pathological immune activation; the phenotypic distributions observed are consistent with the expected trajectory of physiological immune remodeling, which provides some reassurance that the signals captured here reflect normal aging rather than disease-driven effects. The sex imbalance in Panel 1 remains a caveat for some of the sex-related analyses. Additionally, this study assessed surface phenotype and proliferation markers only; functional readouts such as cytokine secretion (IFN-γ, TNF-α) and cytotoxicity (CD107a, granzyme B) were not included. The functional consequences of the phenotypic changes described here therefore remain to be directly established. Finally, serum immunoglobulin levels were not measured, which prevents us from determining whether the late-stage B cell phenotypic changes observed in our oldest participants are accompanied by corresponding shifts in antibody class distribution or total immunoglobulin output.

## 5 Conclusion

Across 146 healthy Chinese adults spanning two to 9 decades of life, we found consistent and largely sex-independent evidence of immune remodeling in all three major lymphocyte compartments. In the T cell pool, CD8^+^ TEMRA accumulation was the dominant signal, accompanied by CD28 loss, naive depletion, and CD57 accumulation in both CD4^+^ and CD8^+^ cells; the parallel decline of CD4^+^ naive cells and CD4^+^CD31^+^ RTE-like cells points to progressive involution of naive T cell generation. NK cells showed age-related contraction of the CD56hiCD16^−^ regulatory subset, expansion of atypical CD56^−^CD16^+^ cells in the oldest participants, and rising PD-1 expression. B cell changes were more modest at younger ages but included IgD^+^CD27^+^ marginal zone-like B cell decline in Panel 1 and switched memory contraction in the older cohort. Together these findings define a multidimensional aging trajectory of the peripheral immune system in healthy Chinese adults, and provide a reference framework for clinical immunophenotyping and vaccine response research.

## Data Availability

The original contributions presented in the study are included in the article/Supplementary Material, further inquiries can be directed to the corresponding authors.
