# Supplementary material for: Age-associated remodeling of NK cell subsets, B cell subsets, and T cell surface markers in peripheral blood of healthy Chinese adults: a prospective multicolor flow cytometry study
Source: Front Aging. 2026 Oct 9;7:1847033. doi: 10.3389/fragi.2026.1847033 (PMC13691342; doi:10.3389/fragi.2026.1847033)
Supplement: Supplementary file 1 [file Table1.docx]

**Supplementary Material**

*Age-Associated Remodeling of NK Cell Subsets, B Cell Subsets, and T Cell Surface Markers in Peripheral Blood of Healthy Chinese Adults: A Prospective Multicolor Flow Cytometry Study*

**Contents:** Supplementary Table 1 (Descriptive statistics); Supplementary Table 2 (Kruskal-Wallis and Dunn post hoc tests); Supplementary Table 3 (Mann-Whitney U test, sex differences); Supplementary Table 4 (Multivariate analysis); Supplementary Table 5 (NK subset segmented Spearman analysis); Supplementary Table 6 (Cross-lineage senescence correlations); Supplementary Table 7 (Immunological Composite Score); Supplementary Figure 1 (Gating strategy); Supplementary Figure 2 (NK piecewise regression); Supplementary Data 1 (Statistical analysis code)

# Supplementary Tables

## Supplementary Table 1A. Descriptive statistics for Panel 1 immunophenotyping parameters (n=103).

*All values are reported as median (IQR). Age groups: young, 20–44 years (n=52); middle-aged, 45–64 years (n=39); older, ≥65 years (n=12).*

| **Parameter** | **Overall** | **Young (20–44)** | **Middle-aged (45–64)** | **Older (≥65)** | **Male** | **Female** | **n** |
| --- | --- | --- | --- | --- | --- | --- | --- |
| **T cell subsets** | | | | | | | |
| CD3+ T cells (% lymphocytes) | 69.30 (62.50–75.70) | 71.95 (64.08–76.08) | 68.30 (62.10–76.00) | 60.35 (42.48–73.03) | 68.45 (62.40–75.52) | 73.20 (63.10–79.30) | 103 |
| CD4+ T cells (% T cells) | 48.40 (40.30–58.10) | 50.45 (43.10–56.23) | 49.60 (42.00–60.90) | 21.40 (18.05–30.20) | 49.30 (42.02–58.65) | 41.10 (21.40–52.50) | 103 |
| CD8+ T cells (% T cells) | 36.40 (30.45–44.65) | 35.75 (32.38–40.58) | 35.00 (23.60–42.55) | 63.55 (45.90–72.53) | 35.85 (28.70–42.45) | 44.50 (35.70–63.60) | 103 |
| CD4+CD57+ (%) | 3.55 (1.02–8.34) | 2.86 (0.74–6.24) | 3.12 (1.23–5.94) | 29.90 (10.86–33.45) | 3.27 (1.06–6.62) | 8.02 (1.01–28.70) | 103 |
| CD8+CD57+ (%) | 25.40 (14.95–38.95) | 20.55 (14.58–29.68) | 26.60 (13.35–42.75) | 45.75 (27.15–56.83) | 25.90 (17.32–39.65) | 18.40 (9.64–33.50) | 103 |
| CD4+Ki67+ (%) | 1.77 (1.20–3.27) | 1.68 (1.02–2.68) | 1.77 (1.31–4.00) | 2.19 (1.57–3.64) | 1.71 (1.20–3.40) | 1.92 (1.53–2.50) | 103 |
| CD8+Ki67+ (%) | 1.66 (0.86–3.04) | 1.35 (0.76–2.69) | 1.85 (1.24–4.46) | 1.42 (0.89–1.79) | 1.71 (0.95–3.56) | 1.51 (0.69–1.89) | 103 |
| CD4+PD-1+ (%) | 24.50 (17.00–32.10) | 24.35 (18.45–30.95) | 28.40 (17.25–32.90) | 15.45 (8.01–28.77) | 24.75 (16.68–32.25) | 22.90 (18.00–28.20) | 103 |
| CD8+PD-1+ (%) | 26.80 (15.65–41.60) | 22.55 (15.05–34.42) | 34.20 (20.30–43.10) | 27.05 (15.15–33.62) | 29.45 (17.43–42.70) | 16.50 (13.80–27.30) | 103 |
| **NK cell subsets** | | | | | | | |
| NK cells (% lymphocytes) | 17.73 (12.71–26.97) | 16.83 (12.27–24.27) | 19.22 (13.88–28.37) | 26.32 (11.18–40.54) | 18.99 (13.82–27.03) | 13.19 (10.49–24.85) | 103 |
| CD56hiCD16− NK (% NK) | 0.75 (0.41–1.51) | 0.99 (0.43–1.72) | 0.57 (0.41–1.31) | 0.48 (0.04–0.89) | 0.74 (0.40–1.37) | 0.78 (0.53–2.21) | 103 |
| CD56dimCD16+ NK (% NK) | 81.70 (75.75–86.60) | 81.80 (77.80–86.45) | 81.00 (73.50–85.95) | 82.00 (77.20–90.12) | 81.75 (75.50–86.75) | 80.80 (78.40–85.90) | 103 |
| CD56−CD16+ NK (% NK) | 7.33 (3.98–10.95) | 8.29 (4.08–11.28) | 7.23 (4.41–11.95) | 3.62 (2.48–7.63) | 7.12 (3.95–10.97) | 7.65 (4.09–9.56) | 103 |
| NK PD-1+ (%) | 1.46 (0.62–4.00) | 1.17 (0.43–3.15) | 2.33 (0.84–4.65) | 2.58 (1.33–3.65) | 1.48 (0.71–4.17) | 0.71 (0.35–2.57) | 103 |
| NK Ki67+ (%) | 2.38 (1.40–5.53) | 2.62 (1.24–4.99) | 2.88 (1.53–7.64) | 1.90 (1.60–2.57) | 2.37 (1.50–5.99) | 2.51 (1.31–3.72) | 103 |
| **B cell subsets** | | | | | | | |
| B cells (% lymphocytes) | 8.73 (6.31–11.95) | 8.91 (6.41–11.22) | 8.69 (6.41–11.65) | 9.21 (3.78–12.65) | 8.71 (6.31–11.28) | 9.78 (7.39–14.00) | 103 |
| Naive B (IgD+CD27−, %) | 9.62 (5.24–14.00) | 8.52 (4.98–13.58) | 11.20 (7.06–14.50) | 5.48 (3.97–10.43) | 10.20 (6.62–14.20) | 5.10 (3.99–9.47) | 103 |
| MZ-like B (IgD+CD27+, %) | 4.60 (2.73–7.55) | 6.08 (2.81–9.33) | 4.28 (2.73–5.78) | 4.47 (1.27–11.15) | 4.59 (2.79–7.03) | 5.14 (2.64–12.50) | 103 |
| Switched memory B (IgD−CD27+, %) | 65.80 (57.65–74.70) | 64.95 (56.48–73.80) | 66.20 (61.70–73.15) | 68.15 (41.30–83.97) | 64.85 (56.70–73.70) | 72.20 (59.40–81.50) | 103 |
| DN B / ABCs (IgD−CD27−, %) | 15.20 (11.30–22.30) | 15.20 (12.10–21.52) | 16.90 (11.65–22.30) | 12.25 (5.30–24.57) | 16.95 (12.35–23.18) | 9.36 (5.60–13.90) | 103 |
| B Ki67+ (%) | 3.15 (1.89–5.19) | 2.94 (1.81–4.86) | 3.53 (2.17–6.96) | 1.93 (0.83–4.86) | 3.52 (1.95–5.61) | 2.31 (1.84–3.32) | 103 |

*IQR, interquartile range; MZ, marginal zone; ABCs, age-associated B cells.*

## Supplementary Table 1B. Descriptive statistics for Panel 2 immunophenotyping parameters (n=43).

*All values are reported as median (IQR). Age groups: young, 20–44 years (n=11); middle-aged, 45–64 years (n=5); older, ≥65 years (n=27). Panel 2 was enriched for older participants.*

| **Parameter** | **Overall** | **Young (20–44)** | **Middle-aged (45–64)** | **Older (≥65)** | **Male** | **Female** | **n** |
| --- | --- | --- | --- | --- | --- | --- | --- |
| **T cell subsets and deep phenotype** | | | | | | | |
| CD4+ T cells (% lymphocytes) | 37.90 (29.90–43.60) | 42.50 (36.50–43.60) | 31.80 (29.10–35.20) | 37.60 (29.65–42.65) | 36.80 (25.70–43.90) | 39.60 (37.30–43.38) | 43 |
| CD8+ T cells (% lymphocytes) | 23.40 (19.45–31.25) | 20.80 (19.05–28.75) | 27.80 (26.60–31.20) | 23.40 (19.75–29.75) | 20.80 (16.80–27.80) | 26.60 (24.45–32.38) | 43 |
| CD4+ naive (CD45RA+CD62L+, %) | 33.50 (18.60–42.50) | 47.70 (38.50–51.70) | 15.30 (12.20–17.90) | 25.70 (18.60–39.60) | 25.70 (17.90–39.50) | 39.60 (27.60–44.78) | 43 |
| CD8+ naive (CD45RA+CD62L+, %) | 22.80 (14.50–32.95) | 40.80 (37.25–50.10) | 17.30 (14.40–18.80) | 20.40 (13.25–26.55) | 21.20 (14.40–28.50) | 26.55 (18.07–37.73) | 43 |
| CD4+ TEMRA (%) | 1.90 (0.65–4.00) | 2.80 (1.90–4.20) | 0.70 (0.30–0.80) | 1.90 (0.60–4.30) | 2.20 (0.70–4.50) | 1.50 (0.72–3.70) | 43 |
| CD8+ TEMRA (%) | 30.20 (22.00–48.45) | 23.40 (15.25–28.20) | 24.66 (22.10–31.40) | 44.10 (27.55–51.59) | 27.40 (21.80–48.30) | 40.30 (28.62–50.10) | 43 |
| CD4+ Memory (CD45RA−, %) | 62.40 (54.90–79.15) | 47.40 (45.40–58.85) | 81.70 (81.50–87.50) | 70.40 (59.20–79.15) | 70.40 (58.20–80.20) | 59.50 (53.10–67.08) | 43 |
| CD8+ Memory (CD45RA−, %) | 38.40 (31.50–45.40) | 31.90 (28.50–38.10) | 53.80 (45.90–58.70) | 38.70 (31.75–45.40) | 42.20 (35.50–48.40) | 33.35 (25.25–39.60) | 43 |
| CD4+CD28+ (%) | 91.20 (87.20–96.10) | 87.20 (85.75–92.15) | 97.30 (92.50–97.30) | 91.10 (88.75–96.10) | 91.60 (88.70–97.20) | 89.70 (87.20–94.75) | 43 |
| CD8+CD28+ (%) | 56.90 (40.95–69.35) | 70.70 (62.10–92.55) | 58.70 (56.90–61.20) | 45.50 (36.15–58.40) | 56.90 (42.20–70.70) | 52.55 (38.32–62.80) | 43 |
| CD4+CD31+ (RTE, %) | 50.50 (42.60–60.55) | 62.30 (54.80–64.85) | 45.00 (39.60–57.00) | 47.00 (38.65–55.90) | 45.50 (38.20–56.60) | 58.45 (49.83–64.95) | 43 |
| CD8+CD31+ (%) | 76.30 (71.15–81.55) | 81.40 (75.60–85.65) | 74.40 (69.60–76.60) | 75.90 (69.60–80.70) | 74.40 (68.50–77.00) | 83.75 (80.70–87.45) | 43 |
| CD4+CD38+ (%) | 37.10 (25.00–51.10) | 51.10 (45.25–54.75) | 24.70 (22.80–29.80) | 30.40 (24.85–44.60) | 32.00 (24.70–48.00) | 45.15 (32.23–51.10) | 43 |
| CD8+CD38+ (%) | 25.70 (16.80–35.00) | 30.40 (26.15–32.15) | 23.20 (14.00–24.60) | 24.70 (16.80–36.05) | 24.60 (15.60–36.70) | 27.80 (23.82–33.05) | 43 |
| CD4+HLA-DR+ (%) | 15.50 (13.00–22.85) | 14.70 (12.45–28.30) | 17.60 (15.50–26.90) | 15.70 (13.35–22.60) | 17.60 (13.70–23.40) | 14.30 (12.32–18.70) | 43 |
| CD8+HLA-DR+ (%) | 37.60 (26.55–48.30) | 23.00 (15.15–29.10) | 51.10 (26.00–51.70) | 42.40 (31.00–49.35) | 40.10 (27.10–48.60) | 30.80 (26.12–41.70) | 43 |
| CD4+CD38+HLA-DR+ (%) | 8.53 (6.03–10.90) | 11.50 (9.04–18.55) | 6.78 (6.04–7.86) | 7.03 (5.78–9.66) | 8.73 (6.04–11.20) | 6.73 (6.04–10.23) | 43 |
| CD8+CD38+HLA-DR+ (%) | 9.39 (5.15–14.25) | 5.26 (2.73–9.62) | 5.81 (5.56–9.86) | 9.98 (6.96–17.15) | 9.39 (5.56–14.70) | 8.27 (4.33–13.23) | 43 |
| CD4+CD57+ (%) | 10.40 (7.37–13.60) | 9.47 (5.31–12.70) | 8.68 (7.42–11.00) | 10.70 (8.06–13.95) | 10.40 (7.80–14.30) | 10.50 (7.35–12.47) | 43 |
| CD8+CD57+ (%) | 34.40 (21.40–48.60) | 29.80 (21.40–37.05) | 29.90 (28.70–33.80) | 41.70 (20.50–51.50) | 34.80 (21.10–54.30) | 33.45 (25.03–41.20) | 43 |
| CD4+Ki67+ (%) | 0.64 (0.41–1.90) | 0.50 (0.29–2.00) | 0.58 (0.38–0.80) | 0.92 (0.46–2.04) | 0.64 (0.38–1.96) | 1.07 (0.46–1.74) | 43 |
| CD8+Ki67+ (%) | 0.59 (0.34–1.36) | 0.40 (0.29–0.82) | 0.36 (0.29–0.80) | 0.90 (0.48–2.85) | 0.80 (0.38–1.30) | 0.48 (0.32–1.40) | 43 |
| CD4+PD-1+ (%) | 41.70 (38.25–48.15) | 42.00 (14.60–47.95) | 53.60 (36.10–59.70) | 41.40 (38.85–45.70) | 40.50 (38.20–48.60) | 42.05 (39.12–45.23) | 43 |
| CD8+PD-1+ (%) | 48.10 (36.30–58.75) | 50.70 (22.15–56.05) | 60.00 (59.50–69.50) | 47.30 (36.30–54.10) | 47.30 (32.60–58.00) | 51.05 (42.30–58.55) | 43 |
| **NK cell subsets** | | | | | | | |
| NK cells (% lymphocytes) | 21.71 (16.17–30.69) | 20.25 (11.48–24.48) | 16.27 (16.07–24.12) | 24.60 (19.73–35.40) | 26.19 (19.97–36.15) | 19.79 (11.81–22.35) | 43 |
| CD56hiCD16− NK (% NK) | 0.58 (0.32–1.02) | 0.99 (0.80–2.10) | 0.50 (0.28–1.23) | 0.41 (0.30–0.81) | 0.50 (0.28–0.98) | 0.80 (0.43–1.29) | 43 |
| CD56dimCD16+ NK (% NK) | 84.70 (77.00–89.65) | 87.00 (84.20–92.20) | 78.50 (74.30–88.20) | 84.30 (77.25–89.25) | 85.70 (79.00–89.90) | 83.05 (68.65–88.68) | 43 |
| CD56−CD16+ NK (% NK) | 9.34 (5.13–15.85) | 4.56 (3.47–10.70) | 6.77 (6.67–17.40) | 11.60 (5.88–15.85) | 8.71 (4.56–13.60) | 12.55 (6.41–24.38) | 43 |
| NK PD-1+ (%) | 4.59 (2.58–5.94) | 4.83 (2.42–6.31) | 3.39 (3.10–5.74) | 4.45 (2.58–5.69) | 4.45 (2.56–5.59) | 5.55 (2.79–6.53) | 43 |
| NK Ki67+ (%) | 1.60 (1.06–2.62) | 0.70 (0.11–1.57) | 1.45 (1.38–1.68) | 1.92 (1.35–3.00) | 1.57 (1.14–2.47) | 1.79 (1.03–2.62) | 43 |
| **B cell subsets** | | | | | | | |
| B cells (% lymphocytes) | 7.47 (4.79–10.55) | 7.46 (6.56–12.35) | 10.30 (7.47–11.00) | 7.21 (3.12–9.71) | 9.24 (4.85–11.00) | 6.79 (4.03–7.93) | 43 |
| Naive B cells (%) | 16.90 (7.25–24.45) | 17.30 (5.16–24.85) | 14.10 (7.14–24.40) | 16.90 (8.84–23.60) | 14.70 (7.36–24.40) | 17.50 (6.22–24.32) | 43 |
| MZ-like B cells (%) | 6.26 (3.29–11.05) | 6.26 (3.74–7.51) | 5.63 (5.31–7.78) | 6.55 (3.00–14.55) | 5.49 (3.46–11.50) | 7.40 (3.25–9.89) | 43 |
| Switched memory B cells (%) | 57.10 (42.70–72.50) | 61.20 (53.80–76.00) | 58.50 (56.10–75.90) | 52.90 (39.40–68.00) | 58.10 (42.50–72.70) | 55.20 (44.67–68.25) | 43 |
| DN B / ABCs (%) | 14.60 (9.25–19.15) | 14.70 (8.79–18.25) | 14.70 (12.40–16.00) | 14.40 (10.09–19.80) | 14.20 (9.21–18.80) | 16.30 (12.47–20.28) | 43 |
| B Ki67+ (%) | 0.75 (0.43–1.94) | 0.68 (0.42–1.20) | 0.68 (0.39–4.23) | 0.80 (0.47–2.81) | 0.68 (0.41–1.30) | 1.05 (0.48–3.44) | 43 |

*TEMRA, terminally differentiated effector memory T cells re-expressing CD45RA; RTE, recent thymic emigrants. Other abbreviations as in Supplementary Table 1A.*

## Supplementary Table 2A. Kruskal-Wallis test and Dunn post hoc comparisons across age groups — Panel 1 (n=103).

*Pairwise Dunn test p values are Bonferroni-corrected. Age groups: young (20–44, n=52); middle-aged (45–64, n=39); older (≥65, n=12). Y, young; M, middle-aged; O, older.*

| **Parameter** | **n** | **Young (20–44) median (IQR)** | **n** | **Middle-aged (45–64) median (IQR)** | **n** | **Older (≥65) median (IQR)** | **KW p** |  | **Y vs M** |  | **Y vs O** |  | **M vs O** |  |
| --- | --- | --- | --- | --- | --- | --- | --- | --- | --- | --- | --- | --- | --- | --- |
| **T cell subsets** | | | | | | | | | | | | | | |
| CD3+ T cells (% lymphocytes) | 52 | 71.95 (64.08–76.08) | 39 | 68.30 (62.10–76.00) | 12 | 60.35 (42.48–73.03) | 0.116 | ns | 1.000 | ns | 0.147 | ns | 0.402 | ns |
| CD4+ T cells (% T cells) | 52 | 50.45 (43.10–56.23) | 39 | 49.60 (42.00–60.90) | 12 | 21.40 (18.05–30.20) | <0.001 | *** | 1.000 | ns | <0.001 | *** | <0.001 | *** |
| CD8+ T cells (% T cells) | 52 | 35.75 (32.38–40.58) | 39 | 35.00 (23.60–42.55) | 12 | 63.55 (45.90–72.53) | <0.001 | *** | 0.954 | ns | <0.001 | *** | <0.001 | *** |
| CD4+CD57+ (%) | 52 | 2.86 (0.74–6.24) | 39 | 3.12 (1.23–5.94) | 12 | 29.90 (10.86–33.45) | <0.001 | *** | 1.000 | ns | <0.001 | *** | <0.001 | *** |
| CD8+CD57+ (%) | 52 | 20.55 (14.58–29.68) | 39 | 26.60 (13.35–42.75) | 12 | 45.75 (27.15–56.83) | 0.003 | ** | 0.282 | ns | 0.002 | ** | 0.083 | ns |
| CD4+Ki67+ (%) | 52 | 1.68 (1.02–2.68) | 39 | 1.77 (1.31–4.00) | 12 | 2.19 (1.57–3.64) | 0.176 | ns | 0.440 | ns | 0.352 | ns | 1.000 | ns |
| CD8+Ki67+ (%) | 52 | 1.35 (0.76–2.69) | 39 | 1.85 (1.24–4.46) | 12 | 1.42 (0.89–1.79) | 0.155 | ns | 0.283 | ns | 1.000 | ns | 0.385 | ns |
| CD4+PD-1+ (%) | 52 | 24.35 (18.45–30.95) | 39 | 28.40 (17.25–32.90) | 12 | 15.45 (8.01–28.77) | 0.098 | ns | 1.000 | ns | 0.179 | ns | 0.164 | ns |
| CD8+PD-1+ (%) | 52 | 22.55 (15.05–34.42) | 39 | 34.20 (20.30–43.10) | 12 | 27.05 (15.15–33.62) | 0.077 | ns | 0.090 | ns | 1.000 | ns | 0.527 | ns |
| **NK cell subsets** | | | | | | | | | | | | | | |
| NK cells (% lymphocytes) | 52 | 16.83 (12.27–24.27) | 39 | 19.22 (13.88–28.37) | 12 | 26.32 (11.18–40.54) | 0.219 | ns | 0.800 | ns | 0.341 | ns | 1.000 | ns |
| CD56hiCD16− NK (% NK) | 52 | 0.99 (0.43–1.72) | 39 | 0.57 (0.41–1.31) | 12 | 0.48 (0.04–0.89) | 0.115 | ns | 0.880 | ns | 0.156 | ns | 0.506 | ns |
| CD56dimCD16+ NK (% NK) | 52 | 81.80 (77.80–86.45) | 39 | 81.00 (73.50–85.95) | 12 | 82.00 (77.20–90.12) | 0.778 | ns | 1.000 | ns | 1.000 | ns | 1.000 | ns |
| CD56−CD16+ NK (% NK) | 52 | 8.29 (4.08–11.28) | 39 | 7.23 (4.41–11.95) | 12 | 3.62 (2.48–7.63) | 0.140 | ns | 1.000 | ns | 0.186 | ns | 0.201 | ns |
| NK PD-1+ (%) | 52 | 1.17 (0.43–3.15) | 39 | 2.33 (0.84–4.65) | 12 | 2.58 (1.33–3.65) | 0.161 | ns | 0.300 | ns | 0.475 | ns | 1.000 | ns |
| NK Ki67+ (%) | 52 | 2.62 (1.24–4.99) | 39 | 2.88 (1.53–7.64) | 12 | 1.90 (1.60–2.57) | 0.249 | ns | 1.000 | ns | 0.628 | ns | 0.410 | ns |
| **B cell subsets** | | | | | | | | | | | | | | |
| B cells (% lymphocytes) | 52 | 8.91 (6.41–11.22) | 39 | 8.69 (6.41–11.65) | 12 | 9.21 (3.78–12.65) | 0.912 | ns | 1.000 | ns | 1.000 | ns | 1.000 | ns |
| Naive B (IgD+CD27−, %) | 52 | 8.52 (4.98–13.58) | 39 | 11.20 (7.06–14.50) | 12 | 5.48 (3.97–10.43) | 0.146 | ns | 0.816 | ns | 0.618 | ns | 0.186 | ns |
| MZ-like B (IgD+CD27+, %) | 52 | 6.08 (2.81–9.33) | 39 | 4.28 (2.73–5.78) | 12 | 4.47 (1.27–11.15) | 0.208 | ns | 0.212 | ns | 1.000 | ns | 1.000 | ns |
| Switched memory B (IgD−CD27+, %) | 52 | 64.95 (56.48–73.80) | 39 | 66.20 (61.70–73.15) | 12 | 68.15 (41.30–83.97) | 0.919 | ns | 1.000 | ns | 1.000 | ns | 1.000 | ns |
| DN B / ABCs (IgD−CD27−, %) | 52 | 15.20 (12.10–21.52) | 39 | 16.90 (11.65–22.30) | 12 | 12.25 (5.30–24.57) | 0.589 | ns | 1.000 | ns | 0.993 | ns | 1.000 | ns |
| B Ki67+ (%) | 52 | 2.94 (1.81–4.86) | 39 | 3.53 (2.17–6.96) | 12 | 1.93 (0.83–4.86) | 0.103 | ns | 0.457 | ns | 0.726 | ns | 0.164 | ns |

**p<0.05; **p<0.01; ***p<0.001; ns, not significant.*

## Supplementary Table 2B. Kruskal-Wallis test and Dunn post hoc comparisons across age groups — Panel 2 (n=43), selected parameters.

*Parameters reaching Kruskal-Wallis significance (p<0.05) or near-significance are shown. Age groups: young (n=11), middle-aged (n=5), older (n=27). Y, young; M, middle-aged; O, older.*

| **Parameter** | **n** | **Young (20–44) median (IQR)** | **n** | **Middle-aged (45–64) median (IQR)** | **n** | **Older (≥65) median (IQR)** | **KW p** |  | **Y vs M** |  | **Y vs O** |  | **M vs O** |  |
| --- | --- | --- | --- | --- | --- | --- | --- | --- | --- | --- | --- | --- | --- | --- |
| **T cell subsets (significant or near-significant parameters)** | | | | | | | | | | | | | | |
| CD4+ naive (CD45RA+CD62L+, %) | 11 | 47.70 (38.50–51.70) | 5 | 15.30 (12.20–17.90) | 27 | 25.70 (18.60–39.60) | <0.001 | *** | <0.001 | *** | 0.002 | ** | 0.376 | ns |
| CD8+ naive (CD45RA+CD62L+, %) | 11 | 40.80 (37.25–50.10) | 5 | 17.30 (14.40–18.80) | 27 | 20.40 (13.25–26.55) | <0.001 | *** | 0.011 | * | <0.001 | *** | 1.000 | ns |
| CD4+ TEMRA (%) | 11 | 2.80 (1.90–4.20) | 5 | 0.70 (0.30–0.80) | 27 | 1.90 (0.60–4.30) | 0.129 | ns | 0.114 | ns | 0.978 | ns | 0.482 | ns |
| CD8+ TEMRA (%) | 11 | 23.40 (15.25–28.20) | 5 | 24.66 (22.10–31.40) | 27 | 44.10 (27.55–51.59) | 0.008 | ** | 1.000 | ns | 0.012 | * | 0.340 | ns |
| CD4+ Memory (CD45RA−, %) | 11 | 47.40 (45.40–58.85) | 5 | 81.70 (81.50–87.50) | 27 | 70.40 (59.20–79.15) | <0.001 | *** | 0.005 | ** | 0.001 | ** | 0.155 | ns |
| CD8+ Memory (CD45RA−, %) | 11 | 31.90 (28.50–38.10) | 5 | 53.80 (45.90–58.70) | 27 | 38.70 (31.75–45.40) | 0.016 | * | 0.010 | ** | 0.264 | ns | 0.196 | ns |
| CD8+CD28+ (%) | 11 | 70.70 (62.10–92.55) | 5 | 58.70 (56.90–61.20) | 27 | 45.50 (36.15–58.40) | 0.009 | ** | 0.422 | ns | 0.013 | * | 0.610 | ns |
| CD4+CD31+ (RTE, %) | 11 | 62.30 (54.80–64.85) | 5 | 45.00 (39.60–57.00) | 27 | 47.00 (38.65–55.90) | 0.004 | ** | 0.082 | ns | 0.004 | ** | 1.000 | ns |
| CD8+HLA-DR+ (%) | 11 | 23.00 (15.15–29.10) | 5 | 51.10 (26.00–51.70) | 27 | 42.40 (31.00–49.35) | 0.002 | ** | 0.345 | ns | 0.001 | ** | 1.000 | ns |
| CD8+CD38+HLA-DR+ (%) | 11 | 5.26 (2.73–9.62) | 5 | 5.81 (5.56–9.86) | 27 | 9.98 (6.96–17.15) | 0.027 | * | 0.959 | ns | 0.026 | * | 1.000 | ns |
| CD4+CD38+HLA-DR+ (%) | 11 | 11.50 (9.04–18.55) | 5 | 6.78 (6.04–7.86) | 27 | 7.03 (5.78–9.66) | 0.029 | * | 0.345 | ns | 0.029 | * | 1.000 | ns |
| **NK cell subsets** | | | | | | | | | | | | | | |
| CD56hiCD16− NK (% NK) | 11 | 0.99 (0.80–2.10) | 5 | 0.50 (0.28–1.23) | 27 | 0.41 (0.30–0.81) | 0.021 | * | 0.577 | ns | 0.016 | * | 1.000 | ns |
| NK Ki67+ (%) | 11 | 0.70 (0.11–1.57) | 5 | 1.45 (1.38–1.68) | 27 | 1.92 (1.35–3.00) | 0.026 | * | 0.663 | ns | 0.027 | * | 1.000 | ns |

**p<0.05; **p<0.01; ***p<0.001; ns, not significant.*

## Supplementary Table 3A. Sex differences in immunophenotyping parameters — Panel 1 (Mann-Whitney U test, n=103; 86 males, 17 females).

| **Parameter** | **Male n** | **Male median (IQR)** | **Female n** | **Female median (IQR)** | **p value** |  |
| --- | --- | --- | --- | --- | --- | --- |
| **Significant parameters (p<0.05)** | | | | | | |
| CD4+ T cells (% T cells) | 86 | 49.30 (42.02–58.65) | 17 | 41.10 (21.40–52.50) | 0.011 | * |
| CD8+ T cells (% T cells) | 86 | 35.85 (28.70–42.45) | 17 | 44.50 (35.70–63.60) | 0.003 | ** |
| CD8+PD-1+ (%) | 86 | 29.45 (17.43–42.70) | 17 | 16.50 (13.80–27.30) | 0.040 | * |
| Naive B (IgD+CD27−, %) | 86 | 10.20 (6.62–14.20) | 17 | 5.10 (3.99–9.47) | 0.028 | * |
| DN B / ABCs (IgD−CD27−, %) | 86 | 16.95 (12.35–23.18) | 17 | 9.36 (5.60–13.90) | <0.001 | *** |
| **Non-significant parameters (selected)** | | | | | | |
| CD3+ T cells (% lymphocytes) | 86 | 68.45 (62.40–75.52) | 17 | 73.20 (63.10–79.30) | 0.647 | ns |
| CD4+CD57+ (%) | 86 | 3.27 (1.06–6.62) | 17 | 8.02 (1.01–28.70) | 0.153 | ns |
| CD8+CD57+ (%) | 86 | 25.90 (17.32–39.65) | 17 | 18.40 (9.64–33.50) | 0.207 | ns |
| NK cells (% lymphocytes) | 86 | 18.99 (13.82–27.03) | 17 | 13.19 (10.49–24.85) | 0.227 | ns |
| CD56hiCD16− NK (% NK) | 86 | 0.74 (0.40–1.37) | 17 | 0.78 (0.53–2.21) | 0.401 | ns |
| NK PD-1+ (%) | 86 | 1.48 (0.71–4.17) | 17 | 0.71 (0.35–2.57) | 0.072 | ns |
| B cells (% lymphocytes) | 86 | 8.71 (6.31–11.28) | 17 | 9.78 (7.39–14.00) | 0.259 | ns |
| Switched memory B (IgD−CD27+, %) | 86 | 64.85 (56.70–73.70) | 17 | 72.20 (59.40–81.50) | 0.057 | ns |

**p<0.05; **p<0.01; ***p<0.001; ns, not significant.*

## Supplementary Table 3B. Sex differences in immunophenotyping parameters — Panel 2 (Mann-Whitney U test, n=43; 29 males, 14 females).

| **Parameter** | **Male n** | **Male median (IQR)** | **Female n** | **Female median (IQR)** | **p value** |  |
| --- | --- | --- | --- | --- | --- | --- |
| **Significant parameters (p<0.05)** | | | | | | |
| CD8+ T cells (% lymphocytes) | 29 | 20.80 (16.80–27.80) | 14 | 26.60 (24.45–32.38) | 0.010 | ** |
| CD8+ Memory (CD45RA−, %) | 29 | 42.20 (35.50–48.40) | 14 | 33.35 (25.25–39.60) | 0.013 | * |
| CD4+CD31+ (RTE, %) | 29 | 45.50 (38.20–56.60) | 14 | 58.45 (49.83–64.95) | 0.015 | * |
| CD8+CD31+ (%) | 29 | 74.40 (68.50–77.00) | 14 | 83.75 (80.70–87.45) | <0.001 | *** |
| NK cells (% lymphocytes) | 29 | 26.19 (19.97–36.15) | 14 | 19.79 (11.81–22.35) | 0.023 | * |
| **Non-significant parameters (selected)** | | | | | | |
| CD8+ TEMRA (%) | 29 | 27.40 (21.80–48.30) | 14 | 40.30 (28.62–50.10) | 0.213 | ns |
| CD8+CD28+ (%) | 29 | 56.90 (42.20–70.70) | 14 | 52.55 (38.32–62.80) | 0.717 | ns |
| CD4+CD28+ (%) | 29 | 91.60 (88.70–97.20) | 14 | 89.70 (87.20–94.75) | 0.407 | ns |
| NK PD-1+ (%) | 29 | 4.45 (2.56–5.59) | 14 | 5.55 (2.79–6.53) | 0.233 | ns |

**p<0.05; **p<0.01; ***p<0.001; ns, not significant.*

## Supplementary Table 4A. Multivariate analysis for significant parameters — Panel 1 (n=103).

*Only parameters significant in univariate Spearman correlation (p<0.05) are shown. Partial Spearman correlations control for sex. OLS regression uses standardized age (z-scored) as the predictor with sex as a covariate; β represents change per SD of age.*

| **Parameter** | **n** | **Spearman r** | **Spearman p** | **Partial r (sex-adj.)** | **Partial p** | **OLS β (std)** | **OLS SE** | **OLS p** | **Age effect indep. of sex** |
| --- | --- | --- | --- | --- | --- | --- | --- | --- | --- |
| **T cell subsets** | | | | | | | | | |
| CD3+ T cells (% lymphocytes) | 103 | −0.256 | 0.009 | −0.258 | 0.009 | −4.087 | 1.009 | <0.001 | Yes |
| CD4+ T cells (% T cells) | 103 | −0.218 | 0.027 | −0.217 | 0.028 | −3.971 | 1.265 | 0.002 | Yes |
| CD4+CD57+ (%) | 103 | +0.338 | <0.001 | +0.337 | <0.001 | 4.615 | 1.007 | <0.001 | Yes |
| CD8+CD57+ (%) | 103 | +0.308 | 0.002 | +0.315 | 0.001 | 5.597 | 1.510 | <0.001 | Yes |
| CD4+Ki67+ (%) | 103 | +0.201 | 0.042 | +0.202 | 0.041 | 0.637 | 0.298 | 0.035 | Yes |
| CD8+PD-1+ (%) | 103 | +0.230 | 0.019 | +0.243 | 0.013 | 3.134 | 1.440 | 0.032 | Yes |
| **NK cell subsets** | | | | | | | | | |
| NK cells (% lymphocytes) | 103 | +0.259 | 0.008 | +0.265 | 0.007 | 3.556 | 0.935 | <0.001 | Yes |
| CD56hiCD16− NK (% NK) | 103 | −0.263 | 0.007 | −0.267 | 0.006 | −0.149 | 0.104 | 0.156 | Yes |
| NK PD-1+ (%) | 103 | +0.256 | 0.009 | +0.266 | 0.007 | 0.624 | 0.387 | 0.110 | Yes |
| **B cell subsets** | | | | | | | | | |
| MZ-like B (IgD+CD27+, %) | 103 | −0.257 | 0.009 | −0.258 | 0.009 | −0.553 | 0.819 | 0.501 | Yes |

*OLS, ordinary least squares; SE, standard error; std, standardized. *p<0.05; **p<0.01; ***p<0.001.*

## Supplementary Table 4B. Multivariate analysis for significant parameters — Panel 2 (n=43).

*Format as in Supplementary Table 4A. Only parameters reaching Spearman significance (p<0.05) are shown. *Switched memory B cells: OLS p=0.052 (borderline).*

| **Parameter** | **n** | **Spearman r** | **Spearman p** | **Partial r (sex-adj.)** | **Partial p** | **OLS β (std)** | **OLS SE** | **OLS p** | **Age effect indep. of sex** |
| --- | --- | --- | --- | --- | --- | --- | --- | --- | --- |
| **CD8+ T cell phenotype** | | | | | | | | | |
| CD8+ naive (CD45RA+CD62L+, %) | 43 | −0.534 | <0.001 | −0.547 | <0.001 | −10.854 | 1.556 | <0.001 | Yes |
| CD8+ TEMRA (%) | 43 | +0.532 | <0.001 | +0.537 | <0.001 | 8.622 | 1.962 | <0.001 | Yes |
| CD8+CD28+ (%) | 43 | −0.552 | <0.001 | −0.551 | <0.001 | −11.610 | 2.915 | <0.001 | Yes |
| CD8+HLA-DR+ (%) | 43 | +0.370 | 0.015 | +0.377 | 0.013 | 7.969 | 1.930 | <0.001 | Yes |
| CD8+CD38+HLA-DR+ (%) | 43 | +0.363 | 0.017 | +0.366 | 0.016 | 3.094 | 1.229 | 0.016 | Yes |
| CD8+Ki67+ (%) | 43 | +0.375 | 0.013 | +0.378 | 0.013 | 0.596 | 0.271 | 0.034 | Yes |
| **CD4+ T cell phenotype** | | | | | | | | | |
| CD4+ naive (CD45RA+CD62L+, %) | 43 | −0.303 | 0.048 | −0.321 | 0.036 | −7.094 | 1.895 | <0.001 | Yes |
| CD4+CD31+ (RTE, %) | 43 | −0.361 | 0.017 | −0.403 | 0.007 | −4.826 | 1.667 | 0.006 | Yes |
| **NK cell subsets** | | | | | | | | | |
| CD56dimCD16+ NK (% NK) | 43 | −0.308 | 0.045 | −0.307 | 0.045 | −2.753 | 1.819 | 0.138 | Yes |
| CD56−CD16+ NK (% NK) | 43 | +0.390 | 0.010 | +0.395 | 0.009 | 2.558 | 1.454 | 0.086 | Yes |
| NK Ki67+ (%) | 43 | +0.420 | 0.005 | +0.420 | 0.005 | 0.781 | 0.337 | 0.026 | Yes |
| **B cell subsets** | | | | | | | | | |
| B cells (% lymphocytes) | 43 | −0.381 | 0.012 | −0.381 | 0.012 | −1.637 | 0.708 | 0.026 | Yes |
| Switched memory B cells (%) | 43 | −0.379 | 0.012 | −0.378 | 0.012 | −5.414 | 2.708 | 0.052 | Yes* |

*OLS, ordinary least squares; SE, standard error. *p<0.05; **p<0.01; ***p<0.001.*

## Supplementary Table 5. Segmented Spearman correlation analysis for NK cell subsets — combined dataset (n=146), breakpoint age 65 years.

*Combined dataset includes Panel 1 (n=103) and Panel 2 (n=43). The formal slope-change test (piecewise linear regression) yielded p=0.094 for CD56−CD16+ NK cells, which did not reach significance.*

| **NK Subset** | **Segment** | **n** | **Spearman r** | **95% CI** | **p value** |  |
| --- | --- | --- | --- | --- | --- | --- |
| CD56−CD16+ NK (% NK) | Overall (n=146) | 146 | +0.133 | [−0.030, 0.290] | 0.109 | ns |
| CD56−CD16+ NK (% NK) | <65 yr | 107 | +0.123 | [−0.068, 0.306] | 0.206 | ns |
| CD56−CD16+ NK (% NK) | ≥65 yr | 39 | +0.482 | [0.196, 0.692] | 0.002 | ** |
| CD56dimCD16+ NK (% NK) | Overall (n=146) | 146 | −0.047 | [−0.208, 0.116] | 0.573 | ns |
| CD56dimCD16+ NK (% NK) | <65 yr | 107 | −0.152 | [−0.332, 0.039] | 0.118 | ns |
| CD56dimCD16+ NK (% NK) | ≥65 yr | 39 | −0.100 | [−0.403, 0.222] | 0.544 | ns |

*95% CI, 95% confidence interval for Spearman r. *p<0.05; **p<0.01; ns, not significant.*

## Supplementary Table 6. Cross-lineage senescence correlations.

*Significant pairwise Spearman correlations between senescence markers from different immune cell compartments. Only statistically significant pairs (p<0.05) are shown.*

| **Marker A** | **Marker B** | **n** | **Spearman r** | **95% CI** | **p value** |  |
| --- | --- | --- | --- | --- | --- | --- |
| **Panel 1 (n=103) — significant cross-lineage pairs** | | | | | | |
| CD4+CD57+ (%) | CD8+CD57+ (%) | 103 | +0.469 | [0.303, 0.607] | <0.001 | *** |
| CD4+CD57+ (%) | CD56hiCD16− NK (%) | 103 | −0.320 | [−0.484, −0.135] | 0.001 | ** |
| CD8+PD-1+ (%) | DN B/ABCs (%) | 103 | +0.294 | [0.107, 0.462] | 0.003 | ** |
| CD8+CD57+ (%) | NK PD-1+ (%) | 103 | +0.288 | [0.100, 0.456] | 0.003 | ** |
| CD4+CD57+ (%) | NK PD-1+ (%) | 103 | +0.279 | [0.090, 0.448] | 0.004 | ** |
| NK PD-1+ (%) | DN B/ABCs (%) | 103 | +0.255 | [0.064, 0.427] | 0.009 | ** |
| CD8+PD-1+ (%) | MZ-like B (%) | 103 | +0.237 | [0.045, 0.411] | 0.016 | * |
| DN B/ABCs (%) | MZ-like B (%) | 103 | +0.196 | [0.003, 0.375] | 0.047 | * |
| **Panel 2 (n=43) — significant cross-lineage pairs** | | | | | | |
| CD8+ TEMRA (%) | CD8+CD28+ (%) | 43 | −0.775 | [−0.872, −0.619] | <0.001 | *** |
| CD8+CD57+ (%) | CD8+CD28+ (%) | 43 | −0.663 | [−0.803, −0.452] | <0.001 | *** |
| CD8+ TEMRA (%) | CD8+CD57+ (%) | 43 | +0.557 | [0.308, 0.734] | <0.001 | *** |
| NK PD-1+ (%) | CD56−CD16+ NK (%) | 43 | +0.327 | [0.029, 0.571] | 0.032 | * |
| CD8+CD28+ (%) | CD56−CD16+ NK (%) | 43 | −0.325 | [−0.570, −0.028] | 0.033 | * |

**p<0.05; **p<0.01; ***p<0.001.*

## Supplementary Table 7. Immunological Composite Score (ICS): construction and summary statistics.

**7A. ICS component parameters.**

*Each parameter was z-scored across all 146 participants and combined with the signed weight below. ICS = Σ (signed z-score × weight).*

| **Feature** | **Direction** | **Weight** |
| --- | --- | --- |
| CD4+CD57+ (%) | Positive | +1 |
| CD8+CD57+ (%) | Positive | +1 |
| CD4+PD-1+ (%) | Positive | +1 |
| CD8+PD-1+ (%) | Positive | +1 |
| NK% (% lymphocytes) | Positive | +1 |
| NK PD-1+ (%) | Positive | +1 |
| CD4% T cells (% T cells) | Negative | −1 |
| CD56hiCD16− NK% (% NK) | Negative | −1 |
| Switched memory B% (% B cells) | Negative | −1 |

**7B. ICS by age group.**

| **Group** | **n** | **Median ICS** | **IQR** |
| --- | --- | --- | --- |
| Young (20–44 years) | 63 | −2.39 | −5.27 to +0.34 |
| Middle-aged (45–64 years) | 44 | −0.78 | −2.50 to +1.82 |
| Older (≥65 years) | 39 | +4.85 | +2.45 to +6.53 |

**7C. Overall statistical summary.**

| **Statistical test** | **Result** |
| --- | --- |
| Overall Spearman r with age (n=146) | r = +0.575, p < 0.001 |
| Kruskal-Wallis H | H = 49.78, p < 0.001 |
| Dunn: Young vs Middle-aged | p = 0.486 (ns) |
| Dunn: Young vs Older | p < 0.001 (***) |
| Dunn: Middle-aged vs Older | p < 0.001 (***) |

*Dunn p values are Bonferroni-corrected. ICS = Immunological Composite Score. ns, not significant.*

# Supplementary Figures

## Supplementary Figure 1. Representative gating strategies for Panel 1 and Panel 2.

*Panel 1 gating hierarchy: FSC/SSC lymphocyte gate → CD45+ singlets → CD3+ T cells (subdivided into CD4+ and CD8+ subsets with CD57 and PD-1 co-staining) → CD3−CD19+ B cells (IgD/CD27 quadrant gating for naive, MZ-like, switched memory, and double-negative subsets) → CD3−CD56+/CD16+ NK cells (classified as CD56hiCD16−, CD56dimCD16+, and CD56−CD16+ subsets). Panel 2 Tube 1 (T cell tube): CD45RA/CD62L quadrant gating within CD4+ and CD8+ gates to define naive (CD45RA+CD62L+), central memory (CD45RA−CD62L+), effector memory (CD45RA−CD62L−), and TEMRA (CD45RA+CD62L−) subsets. Panel 2 Tube 2 (B/NK tube): same as Panel 1.*

**Supplementary Figure 2. Piecewise linear regression for NK cell subsets versus age — combined dataset (n=146), breakpoint at age 65 years.**

*Six NK cell parameters (CD56dimCD16+, CD56−CD16+, CD56hiCD16−, NK PD-1+, NK Ki67+, NK cells % lymphocytes) are shown as scatter plots with piecewise linear regression lines fitted separately for age <65 years (dashed) and age ≥65 years (solid). Blue circles, Panel 1 (n=103); red circles, Panel 2 (n=43). Overall Spearman r, p value, slope-change test p value (p(slope Δ)), and segment-specific slopes (β, per year) are annotated in each subpanel.*

# Supplementary Data

## Supplementary Data 1. Python statistical analysis code.

The complete Python script used for all statistical analyses is provided as a separate plain text file (statistics_analysis.py). The script was executed in Python 3.10 with the following packages: pandas (v2.x), scipy (v1.x), pingouin (v0.5.x), numpy, openpyxl. Input data file: available from the corresponding author upon request.

The script generates all statistical output tables reported in the main manuscript and Supplementary Tables 1–7, including Spearman rank correlations with 95% confidence intervals, Kruskal-Wallis tests with Dunn post hoc comparisons, Mann-Whitney U tests for sex differences, partial Spearman correlations and OLS regression for multivariate analysis, NK subset segmented Spearman analysis, cross-lineage correlation matrices, and Immunological Composite Score construction and statistics.
